# A Thiourea-Based Rotaxane Catalyst: Nucleophilic Fluorination Phase-Transfer Process Unlocked by the Mechanical Bond

**DOI:** 10.1021/acs.orglett.5c00411

**Published:** 2025-03-18

**Authors:** Julio Puigcerver, Juan S. Dato-Santiago, Mateo Alajarin, Alberto Martinez-Cuezva, Jose Berna

**Affiliations:** † Departamento de Química Orgánica, Facultad de Química, Regional Campus of International Excellence “Campus Mare Nostrum”, 16751 Universidad de Murcia, E-30100 Murcia, Spain

## Abstract

We report a five-component clipping approach using activated isophthaloyl-derived esters to synthesize an amide-based thiourea rotaxane. This method overcomes acyl chloride limitations with nucleophilic thiourea threads. The steric hindrance of the mechanical bond enables, for the first time, an interlocked thiourea as a hydrogen-bonding phase-transfer organocatalyst in nucleophilic fluorinations. This highlights how mechanical bonds expand thiourea catalysis to processes previously incompatible with conventional catalysts.

## Graphical abstract

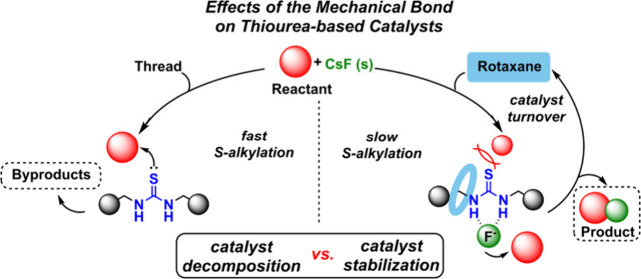

Mechanically interlocked molecules (MIMs) have gained broad interest over the past several decades.^1^ Since Edel Wasserman’s first [2]­catenane synthesis^2^ and Ian T. and Shuyen Harrison’s rotaxane synthesis^3^ via a “statistical approach”, significant efforts have focused on developing robust, high-yielding MIM assembly methods.^4,5^ Leigh-type amide-containing [2]­rotaxane synthesis requires a suitable thread able to template the macrocycle formation through a five-component (clipping) reaction with *p*-xylylenediamine and a diacyl dichloride (Figure 1a).^6^ The functional groups at the thread must be compatible with basic conditions (Et_3_N) and highly reactive acyl chlorides. Thus, any nucleophilic functionality that could react with acyl chlorides, leading to unwanted byproducts, should be avoided.

**Figure 1:**
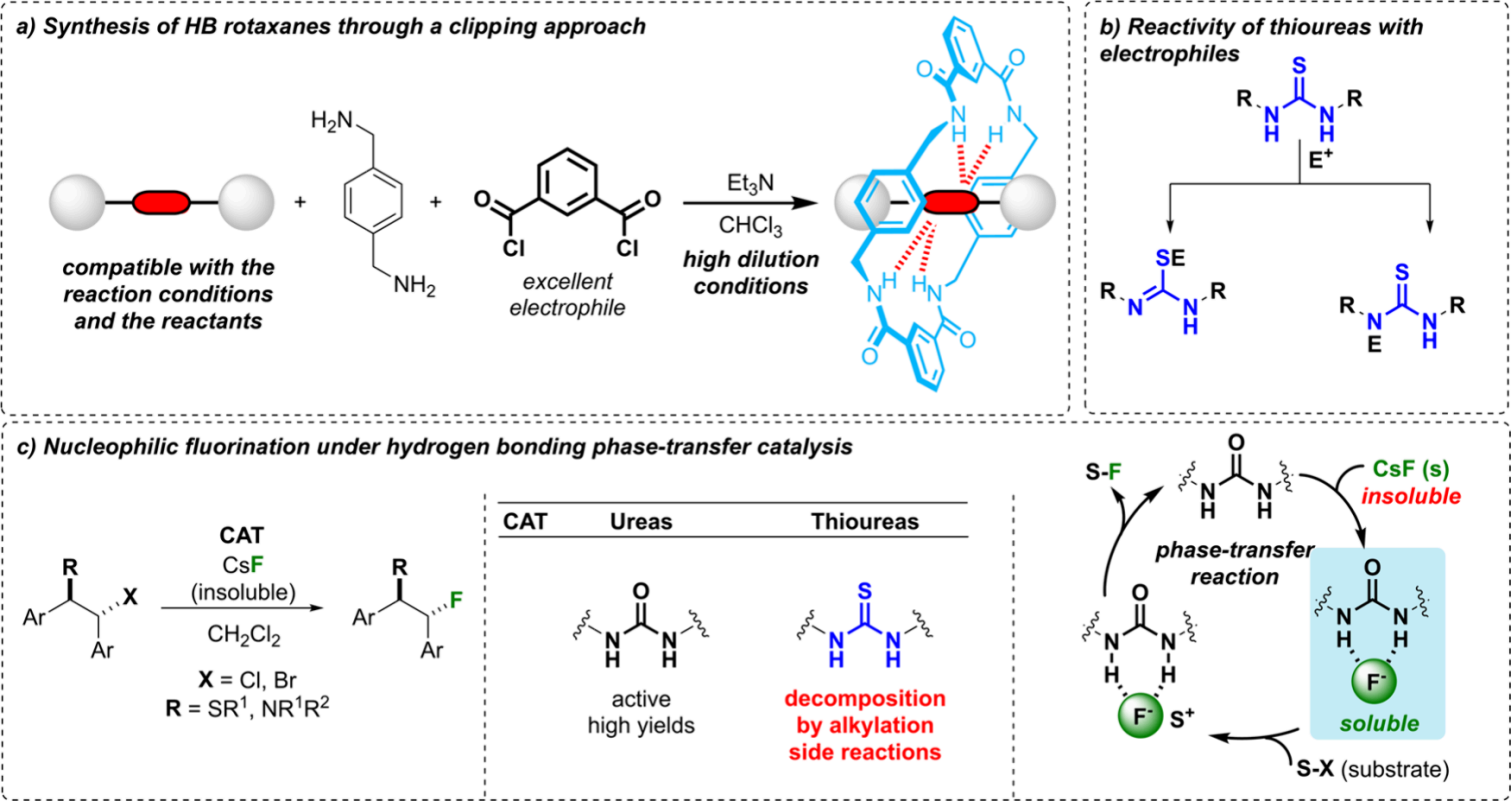
(a) Synthesis of Leigh-type hydrogen-bonded [2]­rotaxanes following a clipping methodology. (b) Nucleophilic addition of thiourea derivatives to electrophiles. (c) Nucleophilic fluorination of diverse electrophiles with CsF under hydrogen-bonding phase-transfer (PT) catalysis.^7,8^

A urea function has been proven to be compatible with these standard reaction conditions, thus allowing the synthesis of hydrogen-bonded (HB) urea-containing rotaxanes, which were further applied as phase-transfer (PT) organocatalysts for nucleophilic fluorination reactions with the insoluble CsF as the fluorine source (Figure 1c).^7,8^ Preliminary theoretical calculations predicted that thiourea-based systems can be used as potential catalysts for this PT process, with an increased activity compared to related ureas.^7a^ Thus, with the aim of using analogous thiourea-containing rotaxanes, assembled through a clipping methodology, as catalysts for this transformation, two challenging scenarios have now been detected. First, thiourea derivatives are known to react with diverse electrophiles, such as alkyl halides or acyl halides, by attack at its sulfur and nitrogen atoms (Figure 1b).^9^ Thus, thiourea-based threads might not be suitable substrates for the assembly of HB rotaxanes by using the readily available isophthaloyl chloride in the five-component clipping approach, and consequently, a synthetic alternative should be proposed.^10^ The second challenge to address is the limitation found by thiourea derivatives in their use as hydrogen-bonding PT catalysts for nucleophilic fluorination reactions, where these systems undergo an undesired process with the electrophilic substrates (Figure 1c).^7a,11^ We planned to overcome this limitation with the aid of the mechanical bond, known to influence the reactivity of functional groups at the threads in various ways.^12^ One of the most common effects of such a bond, often undesired, is the reduced reactivity of functions located within the macrocycle cavity, caused by the sterically crowded environment,^13^ highlighting the decrease in the nucleophilicity of diverse amines^14^ or the on–off switching of interlocked organocatalysts.^15^ More intriguingly, recent studies have revealed that the mechanical bond can facilitate reactions that do not occur in its absence,^16^ thus enhancing the potential applications of interlocked architectures in synthesis and catalysis.^17^

Herein, we present for the first time the use of activated isophthaloyl-derived esters for the assembly of HB rotaxanes by following a clipping approach, being a suitable alternative to the employment of more reactive acyl chlorides.^18^ Thus, we successfully assembled a thiourea-based rotaxane, which was shown to be an efficient PT catalyst in fluorination reactions. The mechanical bond allows the catalysis by sterically hindering the thiourea function, thus weakening its ability to react with the electrophilic substrates present in the media.

In our hands, the five-component reaction of tetrabutylfumaramide **1**, selected as a model thread,^19^ with isophthaloyl chloride (**3a**) and *p*-xylylenediamine (**4**) afforded rotaxane **2** in 71% yield (Table 1, entry 1).^20^ Under identical conditions, we tested activated isophthaloyl-derived esters synthesized from pentafluorophenol (**3b**), *p*-nitrophenol (**3c**), and *N*-hydroxysuccinimide (**3d**) as potential macrocycle precursors, also evaluating the effect of Et_3_N (see the Supporting Information for synthetic procedures). Similar pentafluorophenyl diesters have been used for macrocyclic tetralactam assembly.^21^ ^1^H NMR analysis of the crude reaction mixture allowed conversion calculation by integration of the fumaramide double bond signals in free thread **1** (δ = 7.37 ppm) and interlocked rotaxane **2** (δ = 5.97 ppm) (Figures S1 and S2). As anticipated, reactions with **3b** yielded rotaxane **2** in 47% yield with Et_3_N, but an only 19% yield without it (Table 1, entries 2 and 3). The release of acidic pentafluorophenol (p*K*_a_ = 5.5) likely disrupts hydrogen bonding between thread **1** and the macrocycle precursors, reducing the templating efficiency. Et_3_N mitigates this possibly forming hydrogen-bonded complexes.^22^ *p*-Nitrophenyl ester **3c** was unreactive, likely due to its poor solubility (Table 1, entries 4 and 5). In contrast, using *N*-hydroxysuccinimide ester **3d**, rotaxane **2** was obtained in 30% yield without Et_3_N. Here, the released *N*-hydroxysuccinimide remains insoluble, making external base addition unnecessary (Table 1, entries 6 and 7). However, adding Et_3_N slightly increases the medium polarity, partially solubilizing *N*-hydroxysuccinimide and interfering with rotaxane formation. During the clipping reactions, esters **3b** and **3d** were nearly consumed, forming the corresponding catenane and free macrocycle, with the latter isolated for catalytic testing. Thus, activated esters such as **3** serve as effective HB rotaxane precursors, offering an alternative to isophthaloyl chlorides, though further optimization is needed for achieving higher yields.

**Table 1:**
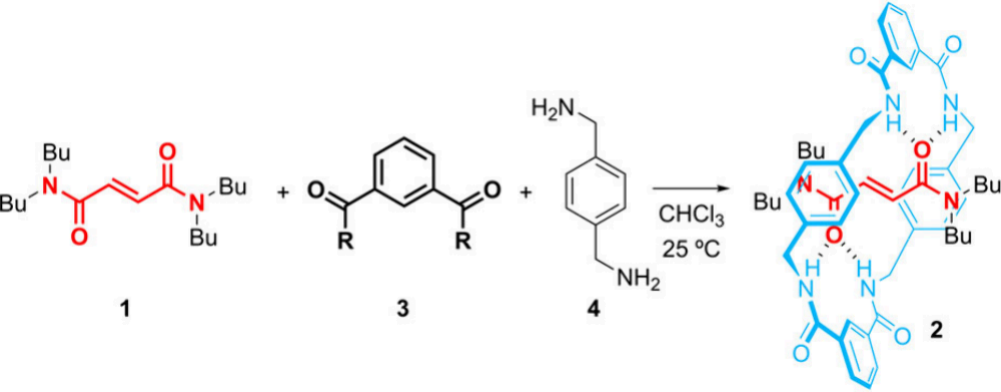

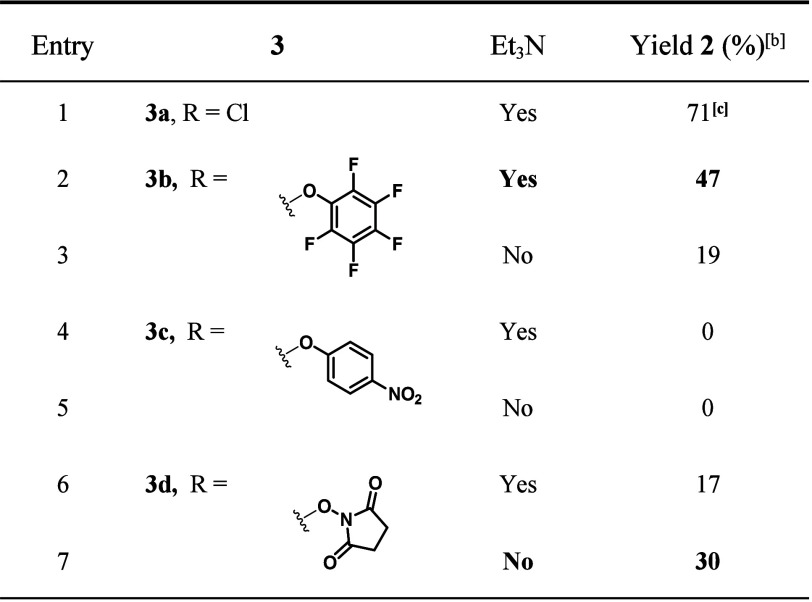
Isophthaloyl Esters **3** for the Synthesis of Rotaxane **2** ^a^

a Thread **1** (0.3 mmol), *p-*xylylenediamine (**2**) (8 equiv), **3** (8 equiv), Et_3_N (24 equiv, when required), CHCl_3_ (200 mL), 25 °C, 4 h.

b Determined by ^1^H NMR from the reaction crude.

c Isolated yield reported in ref 20.

We next synthesized thread **5**, including a thiourea moiety as the catalytic active site and a glycylglycine (GlyGly) template for driving the formation of the tetralactam ring (Scheme 1; see the Supporting Information for full synthetic details). As expected, when thread **5** was submitted to the standard five-component clipping conditions with isophthaloyl chloride (**3a**), the formation of rotaxane **6** was neither observed nor detected by HRMS or ^1^H NMR in the reaction crude. Instead, many unidentified byproducts were formed, with total consumption of **5**. Fortunately, the employment of activated esters **3b** and **3d** allowed us to isolate the desired rotaxane **6**. The reaction with ester **3b**, in the presence of Et_3_N, yielded 25% rotaxane **6**, whereas with *N*-hydroxysuccinimide-derived ester **3d**, a lower conversion was observed (without Et_3_N). Moreover, unreacted thread **5** was fully recovered in both reactions, proving its stability under these conditions. The structure of rotaxane **6** was confirmed by single-crystal X-ray diffraction (Scheme 1, inset).^23^ In the solid state, the macrocycle sits over the GlyGly binding site, forming hydrogen bonds with its two oxygen atoms and one NH amide group. Additionally, the thiourea NH protons interact intermolecularly with a neighboring macrocycle’s oxygen, forming bifurcated hydrogen bonds (see Figure S7).

**Scheme 1:**
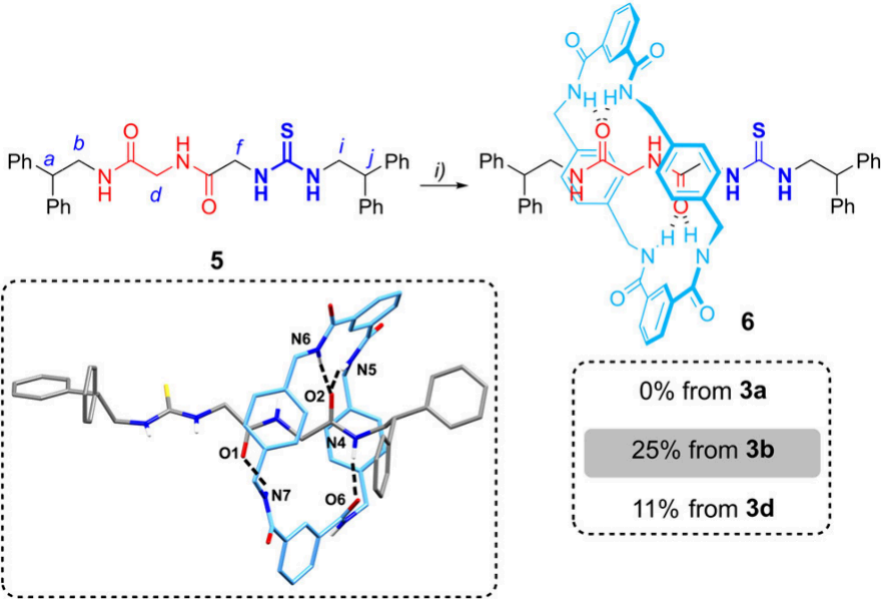
Synthesis of Thiourea-Based Rotaxane **6** from Thread **5** (inset, X-ray structure of rotaxane **6**)­^a^

a Reaction conditions: (i) *p*-xylylenediamine (8 equiv), isophthaloyl dichloride **3a** or activated ester **3b** or **3d** (8 equiv), Et_3_N (24 equiv, for **3b**), CHCl_3_, 25 °C, 4 h. Intramolecular hydrogen-bond lengths (Å) (angles (deg)): N6–H6···O2, 3.10 (175.1); N5–H5···O2, 3.08 (167.3); N4–H4···O6, 2.84 (167.6); N7–H7···O1, 2.97 (141.3). For the sake of clarity, selected hydrogens and solvent molecules have been omitted.

We then tested thread **5** and rotaxane **6** as PT catalysts for the nucleophilic fluorination reaction of substrate **7** in the presence of CsF, insoluble in the reaction media (Scheme 2).^24^ As previously reported for other thioureas,^7a^ thread **5** rapidly reacted with electrophilic substrate **7**, giving rise to a mixture of products and only detecting the desired fluorinated compound **8** in a low 8% yield. In stark contrast, rotaxane **6** was able to efficiently catalyze the fluorination reaction, triggering the formation of **8** in a remarkable 82% yield. The analysis of the reaction crude allowed us also to identify compound **9**, presumably formed by the sulfur alkylation of catalyst **6** with the halides, followed by elimination of pyrrolidine. We followed the alkylation reactions by ^1^H NMR of thread **5** and rotaxane **6** with halide **7**, finding that interlocked thiourea **6** was more stable than free thread **5**, due to the steric hindrance exerted by the tetrabenzylic amide macrocycle (Figure 2a).^13^ While thread **5** smoothly reacted with halide **7** in 4 h, giving various compounds, rotaxane **6** was partially stable even at 24 h (see Figures S3 and S4). After 48 h, the complete consumption of **6** was detected, and we were able to isolate compound **9** in 85% yield (Figure 2b), which was proven to be an inactive species in the fluorination of substrate **7** (10% conversion in 24 h (see entry 4 of Table S1)).

**Scheme 2:**
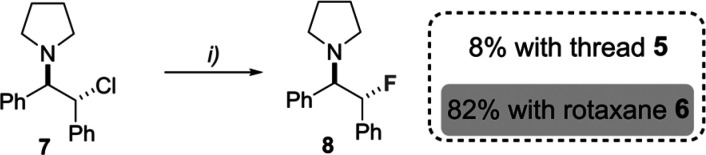
Fluorination Reaction of Compound **7** Catalyzed by Thiourea-Based Thread **5** or Its Corresponding Rotaxane **6** ^a^

a Reactions conditions: (i) CsF (1.2 equiv), catalyst **5** or **6** (10 mol %), CD_2_Cl_2_ (0.25 M), 25 °C, 24 h.

**Figure 2:**
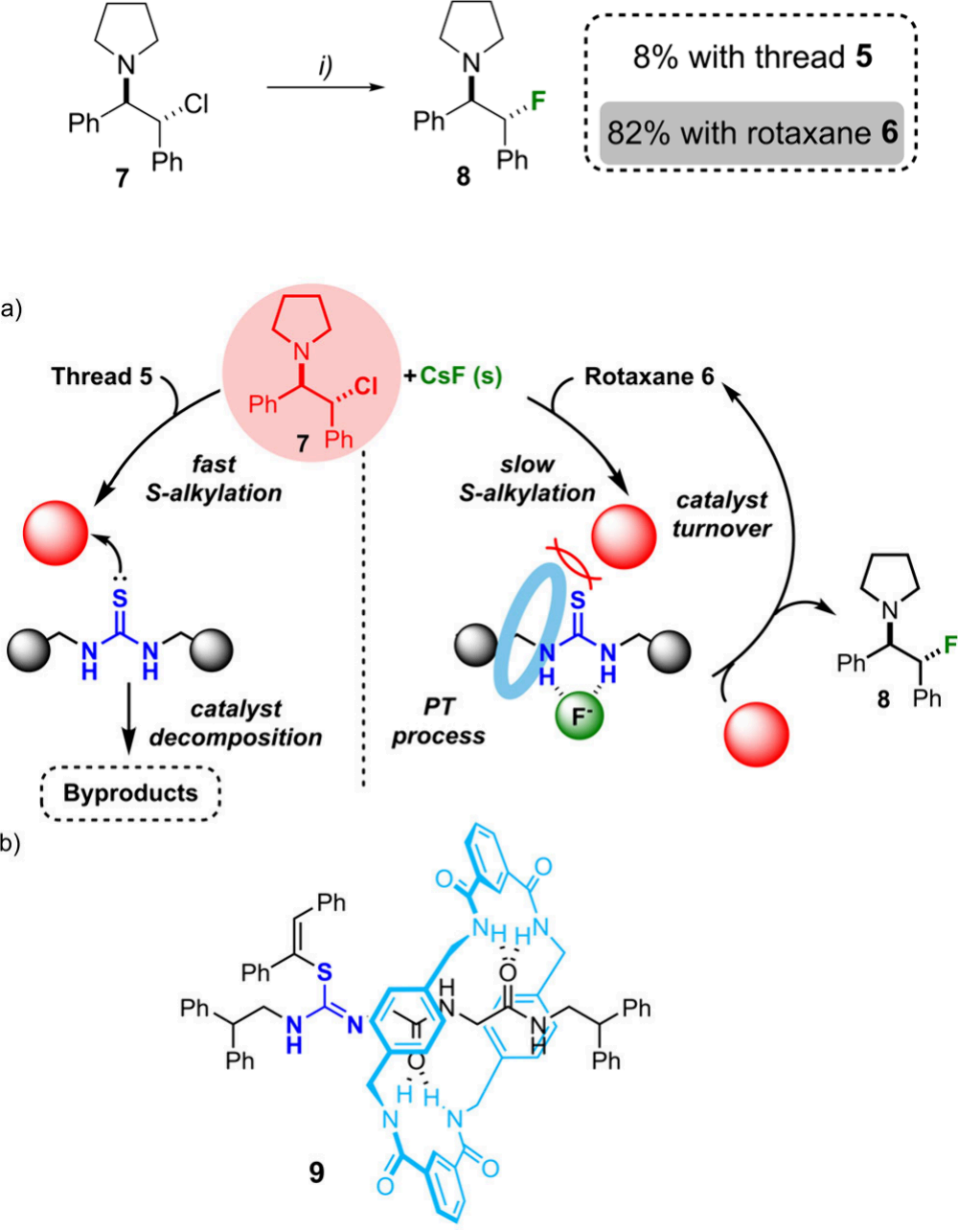
(a) Fluorination of **7** catalyzed by rotaxane **6** and degradation of thread **5**. (b) Compound **9** isolated after the slow degradation of catalyst **6**.

The ^1^H NMR analysis of thread **5** and rotaxane **6** in deuterated chloroform shows that the macrocycle primarily interacts with the GlyGly template (Figure S5 and Table S2), consistent with the solid-state observations. While most thread signals are shielded by the entwined macrocycle, GlyGly methylene protons H_d_ and H_f_ (see Scheme 1 for lettering) undergo significant shifts to higher fields (Δδ = −0.96 and −0.51 ppm), similar to related GlyGly rotaxanes.^25^ Upon addition of TBAF (up to 2 equiv), the macrocycle shifts toward the thiourea function (Figure S6). The thiourea NH groups bind fluoride, causing line broadening, while the macrocycle’s amide NHs shift significantly, indicating cooperative anion interaction. Notably, fluoride binding alters GlyGly methylene signals (H_d_, Δδ = 0.53 ppm, deshielded; H_f_, Δδ = −0.46 ppm, shielded), showing that the macrocycle hydrogen bonds with thiourea, blocking it and reducing its nucleophilicity.^26^ The shielding exerted by the mechanical bond prevents catalyst **6** from rapid decomposition, maximizing CsF solubility and enabling catalyst turnover during fluorination. Interestingly, fluorination does not proceed without a PT catalyst or with the free tetraamide macrocycle (Table S1), highlighting the impact of mechanical bonding in organocatalysis. These experiments prove that thiourea **6** is required in order to solubilize the fluoride source. Gouverneur and co-workers proposed that this process begins with the self-ionization of compound **7** to form the aziridinium–chloride ion pair, which was further stabilized by hydrogen bonding by the hydrogen-bonding catalyst,^7^ although an initial chloride abstraction by the thiourea could also be feasible.^27^ Then an anion exchange happened, in this case, with the fluoride solubilized by the phase-transfer thiourea catalyst, followed by a nucleophilic attack on the aziridinium intermediate, selectively forming fluorinated compound **8** with retention of the initial configuration.

In summary, we successfully employed activated isophthaloyl-derived esters as precursors for the assembly of HB amide-based rotaxanes by following a clipping approach. Although the overall yields obtained with these esters were generally lower than those achieved with the chloride derivatives, this method offers a significant advantage. It enables the formation of HB rotaxanes with threads incompatible with the well-known standard reaction conditions. This advantage was demonstrated in the case of thiourea-containing threads, which possess pronounced nucleophilic character and are incompatible with acyl chloride derivatives. While isophthaloyl chloride led to the formation of several unidentified byproducts, the use of isophthaloyl esters effectively allowed the formation of the desired interlocked thioureas, avoiding undesirable side reactions. These findings address the critical challenge of compatibility between different precursors in the synthesis of mechanically interlocked molecules.

Remarkably, the mechanical bond in the interlocked thiourea facilitates its application as a phase-transfer catalyst in nucleophilic fluorination reactions, a process limited to urea-containing catalysts.^7,8^ Free thioureas tend to decompose under such reaction conditions, whereas the steric hindrance exerted by the mechanical bond minimizes thiourea’s reactivity toward electrophiles. This steric effect not only temporarily preserves the integrity of the thiourea but also ensures the catalyst turnover, unlocking a new reactivity for thiourea-based catalysts.

## Supplementary Material



## Data Availability

The data underlying this study are available in the published article and its Supporting Information.

## Notes

Supplemental experimental procedures, Figures S1–S6, Tables S1–S4, X-ray refinement data, and supplemental references (PDF)
