# Supplementary material for: A Thiourea-Based Rotaxane Catalyst: Nucleophilic Fluorination Phase-Transfer Process Unlocked by the Mechanical Bond
Source: Org Lett. 2025 Mar 18;27(12):2873–8. doi: 10.1021/acs.orglett.5c00411 (PMC12128099; doi:10.1021/acs.orglett.5c00411)
Supplement: Supplementary file 1 [file ol5c00411_si_001.pdf]

SUPPORTING INFORMATION

A Thiourea-based Rotaxane Catalyst: Nucleophilic Fluorination Phase-Transfer Process Unlocked by the Mechanical Bond

Julio Puigcerver,[a] Juan S. Dato-Santiago,[a] Mateo Alajarin,[a] Alberto Martinez-Cuezva,[a,*] Jose Berna[a,*]

[a] Departamento de Química Orgánica, Facultad de Química, Regional Campus of International Excellence "Campus Mare Nostrum", Universidad de Murcia, E-30100 Murcia, Spain.

Table of Contents ……………………………………………………………………………………

1. General experimental section …………………………………………………………………… **S1**

2. Synthesis of fumaramide-based thread 1 ……………………………………………………… **S2**

3. Synthesis of isophthaloyl-derived esters 3b-d ………………………………………………… **S2**

4. Synthesis of fumaramide-based rotaxane 2 with isophthaloyl derivatives 3 as precursors … **S3**

5. Synthesis of thiourea-based thread 5 …………………………………………………………… **S6**

6. Failed synthesis of thiourea-based rotaxane 6 by using isophthaloyl dichloride (3a) ……… **S8**

7. Synthesis of thiourea-based rotaxane 6 by using activated esters 3b and 3d as precursors … **S8**

8. Reaction of thiourea-based thread 5 and rotaxane 6 with substrate 7 ……………………… **S10**

9. Phase-transfer nucleophilic fluorination of substrate 7 ……………………………………… **S13**

10. Stacked ^{1}H NMR spectra of thread 5 and rotaxane 6 ………………………………………… **S14**

11. Stacked ^{1}H NMR spectra of rotaxane 6 with increasing amounts of TBAF ………………… **S15**

12. Crystal data and structure refinements for thiourea-based rotaxane 6 …………………… **S16**

13. ^{1}H, ^{13}C and ^{19}F NMR Spectra of synthesized compounds …………………………………… **S18**

14. References …………………………………………………………………………………… **S33**

1. General experimental section

Unless stated otherwise, all reagents were purchased from Aldrich Chemicals and used without further purification. HPLC grade solvents (Scharlab) were nitrogen saturated and were dried and deoxygenated using an Innovative Technology Inc. Pure-Solv 400 Solvent Purification System. Column chromatography was carried out using silica gel (60 Å, 70-200 µm, SDS) as stationary phase, and TLC was performed on precoated silica gel on aluminium cards (0.25 mm thick, with fluorescent indicator 254 nm, Fluka) and observed under UV light. All melting points were determined on a Kofler hot-plate melting point apparatus and are uncorrected. ^{1}H- and ^{13}C-NMR spectra were recorded on a Bruker Avance 300, 400 and 600 MHz instruments. ^{1}H NMR chemical shifts are reported relative to Me_4Si and were referenced via residual proton resonances of the corresponding deuterated solvent, whereas ^{13}C NMR spectra are reported relative to Me_4Si using the carbon signals of the deuterated solvent. Signals in the ^{1}H and ^{13}C NMR spectra of the synthesized compounds were assigned with the aid of DEPT, APT, or two-dimensional NMR experiments (COSY, NOESY, ROESY, HMQC and HMBC). Abbreviations of coupling patterns are as follows: br, broad; s, singlet; d, doublet; t, triplet; q, quadruplet; qui, quintuplet; m, multiplet. The deuterated solvent $CDCl_3$ was dried over $CaCl_2$ and stored with molecular sieves prior to use. Coupling constants (*J*) are expressed in Hz. High-resolution mass spectra (HRMS) were obtained using a time-of-flight (TOF) instrument equipped with electrospray ionization (ESI).

Abbreviation list:

HPLC: High Performance Liquid Chromatography

NMR: Nuclear Magnetic Resonance

IR: Infrared

TLC: Thin Layer Chromatography

UV: Ultraviolet

DCC: *N,N'*-dicyclohexylcarbodiimide

EDCI·HCl: *N*-(3-dimethylaminopropyl)-*N*'-ethylcarbodiimide hydrochloride

HOBt: hydroxybenzotriazole

DMAP: 4-dimethylaminopyridine

TFA: trifluoroacetic acid

THF: tetrahydrofurane

DMSO: dimethyl sulfoxide

2. Synthesis of fumaramide-based thread 1

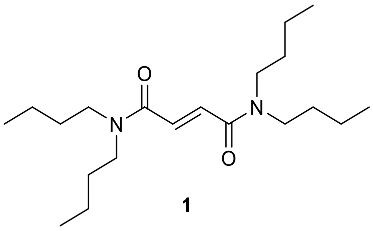

1

Thread **1** was synthesized following a described procedure previously reported in literature and showed identical spectroscopic data as those reported therein.[1]

3. Synthesis of isophthaloyl-derived esters 3b-d

Ester 3b

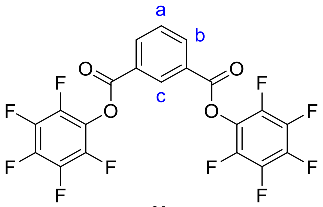

3b

In a 500 mL flask, isophthaloyl chloride (1.51 g, 7.44 mmol) was added to a solution of pentafluorophenol (2.08 mL, 19.23 mmol) in CH_2Cl_2 (200 mL) under a nitrogen atmosphere at 0 °C (ice bath). Then Et_3N (2.67 mL, 19.21 mmol) was added dropwise, and DMAP portion wise (10.0 mg). The reaction mixture was stirred for 24 hours at 25 ºC. After this time, the solvent was removed using a rotary evaporator. The resulting crude was purified by column chromatography using silica gel as the stationary phase and a mixture of hexane/ethyl acetate (7:1) as the eluent, to give the title product as a white solid (3.31 g, 90%). Compound **3b** showed identical physical and spectroscopic data as those reported in the literature.[2]
^{1}H-NMR (300 MHz, $CDCl_3$) δ = 9.02 (t, J = 1.7 Hz, 1H, H_c), 8.53 (dd, J = 1.7, 7.9 Hz, 2H; H_b), 7.79 (t, J = 7.9 Hz, 1H, H_a) ppm; **^{19}F-NMR** (282 MHz, $CDCl_3$) δ = -152.20 – -152.40 (m), -157.04 (t, J = 21.7 Hz), -161.64 – -161.62 (m) ppm.

Ester 3c

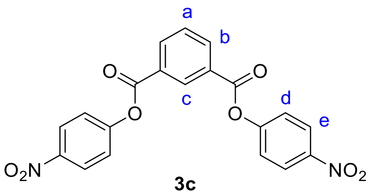

3c

In a 250 mL flask, isophthaloyl chloride (1.36 g, 6.70 mmol) was added to a solution of *p*-nitrophenol (2.55 g, 18.33 mmol) in CH_2Cl_2 (200 mL) under a nitrogen atmosphere at 0 °C (ice bath). Then Et_3N (2.55 mL, 18.31 mmol) was added dropwise, and DMAP portion wise (10.0 mg). The reaction mixture was stirred for 24 hours at 25 ºC. After this time, the white solid was filtered, washed with $CH_2Cl_2$and dried under vacuum

(**3c**, 2.29 g, 84%). Compound **3c** showed identical physical and spectroscopic data as those reported in the literature.[3]

^{1}H-NMR (300 MHz, DMSO-d_6, 345 K) δ = 8.81 (t, J = 1.6 Hz, 1H, H_c), 8.48 (dd, J = 1.8, 7.8 Hz, 2H, H_b), 8.35-8.28 (m, 4H, H_e), 7.86 (t, J = 7.8 Hz, 1H, H_a), 7.66-7.60 (m, 4H, H_d) ppm.

Ester 3d

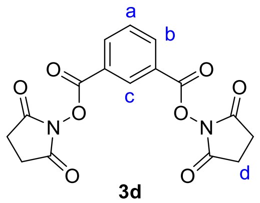

3d

In a 500 mL flask, isophthalic acid (3.00 g, 18.06 mmol) was added to a solution of *N*-hydroxysuccinimide (4.57 g, 39.73 mmol) in anhydrous THF (300 mL) at 0 °C (ice bath). Then, DCC (8.2 g, 39.73 mmol) was added. The reaction mixture was stirred for 24 hours at 25 ºC. After this time, the solvent was removed using a rotary evaporator. The resulting solid was dissolved in CH_2Cl_2 (200 mL) and the solution washed with H_2O (4 x 100 mL). The organic phase was dried over $MgSO_4$, filtered and the solvent removed using a rotary evaporator. The resulting solid was recrystallized in isopropanol to yield a white solid (**3d**, 3.69 g, 57%). Compound **3d** showed identical physical and spectroscopic data as those reported in the literature.[4]

^{1}H-NMR (300 MHz, $CDCl_3$) δ = 8.90 (t, J = 1.7 Hz, 1H, H_c), 8.44 (dd, J = 1.7, 7.9 Hz, 2H, H_b), 8.44 (t, J = 7.9 Hz, 1H, H_a), 2.93 (s, 8H, H_d) ppm.

4. Synthesis of fumaramide-based rotaxane 2 with isophthaloyl derivatives 3 as precursors

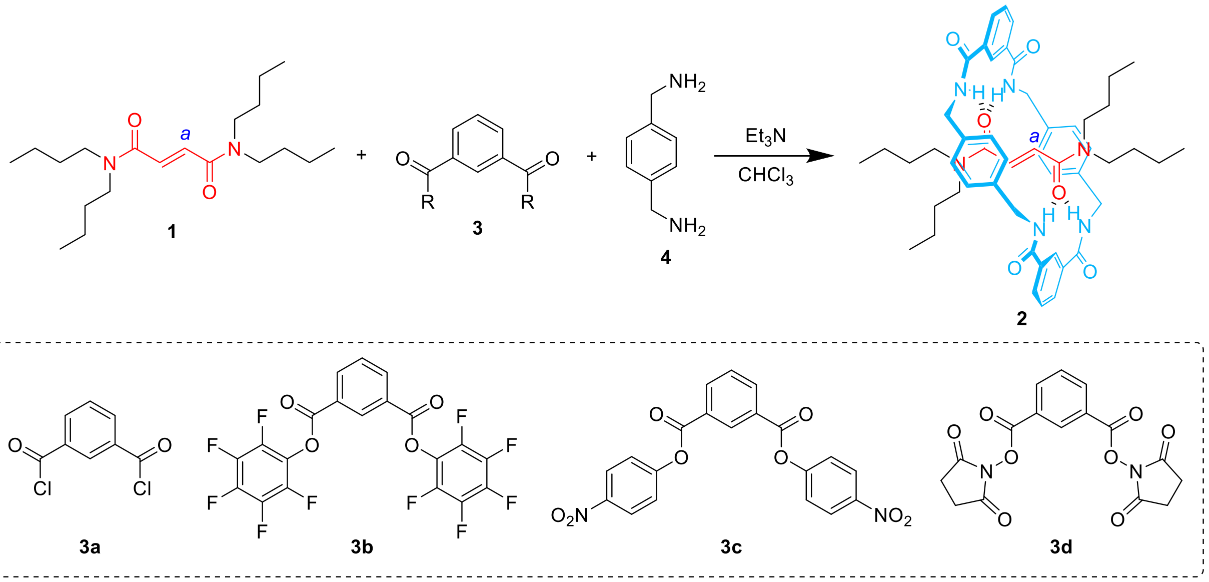

Scheme S1. *Reactions conditions*: *p*-xylylenediamine (**4**) (8 equiv), isophthaloyl derivative **3** (8 equiv), triethylamine, *if required* (24 equiv), $CHCl_3$, 25 ºC.

Thread **1** (1 equiv) and Et_3N (24 equiv, *if required*) in anhydrous $CHCl_3$ (500 mL/mmol of thread) were stirred vigorously whilst solutions of *p*-xylylenediamine (**4**) (8 equiv) in anhydrous $CHCl_3$ (20 mL) and the corresponding isophthaloyl derivative **3** (8 equiv.) in anhydrous $CHCl_3$ (20 mL) were simultaneously added (0.06 mL/min) for 5-6 h using motor-driven syringe pumps. After 24 h, the resulting suspension was filtered through a Celite® pad, and the filtrate washed with water (2 x 50 mL), a solution of HCl 1M (2 x 50 mL), a saturated solution of $NaHCO_3$ (2 x 50 mL) and brine (2 x 50 mL). The organic phase was dried over $MgSO_4$ and the solvent removed under reduced pressure. The yield of the assembled rotaxane **2** for each reaction was calculated by ^{1}H NMR (400 MHz, $CDCl_3$), by integrating the signal related to the remaining thread **1** (H_a) and that related to the formed rotaxane **2**, following the equation:

Yield **2** (%) = (integral **2** / integral **2**+**1**) x 100

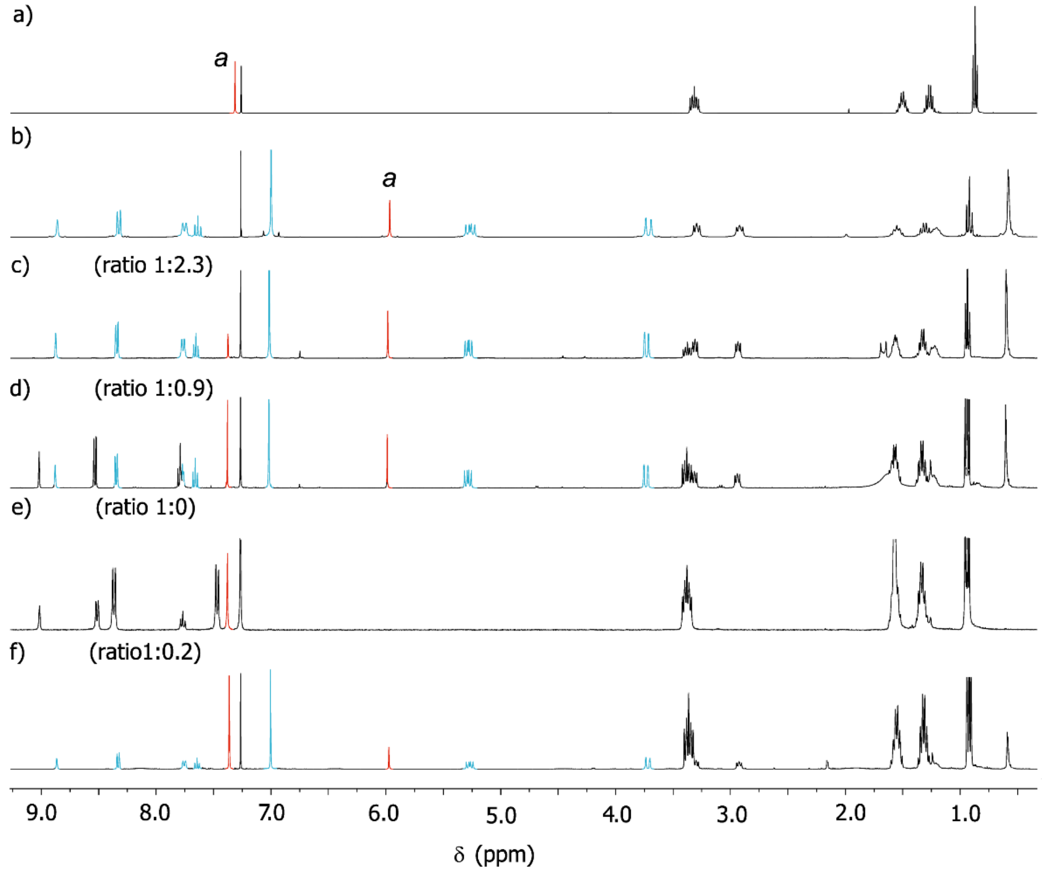

Figure S1. Stack of ^{1}H NMR spectra (400 MHz, $CDCl_3$) of: a) thread **1**; b) isolated rotaxane **2**; c) crude reaction with isophthaloyl chloride **3a**; d) crude reaction with pentafluorophenyl-derived ester **3b**; e) crude reaction with *p*-nitrophenyl-derived ester **3c**; f) crude reaction with *N*-hydroxysuccinimide-derived ester **3d**. Ratios calculated with the integral of the signal related to proton H_a (colored in red) for thread **1** and rotaxane **2**. *All the reactions were carried out in the presence of Et_3N (24 equiv).*

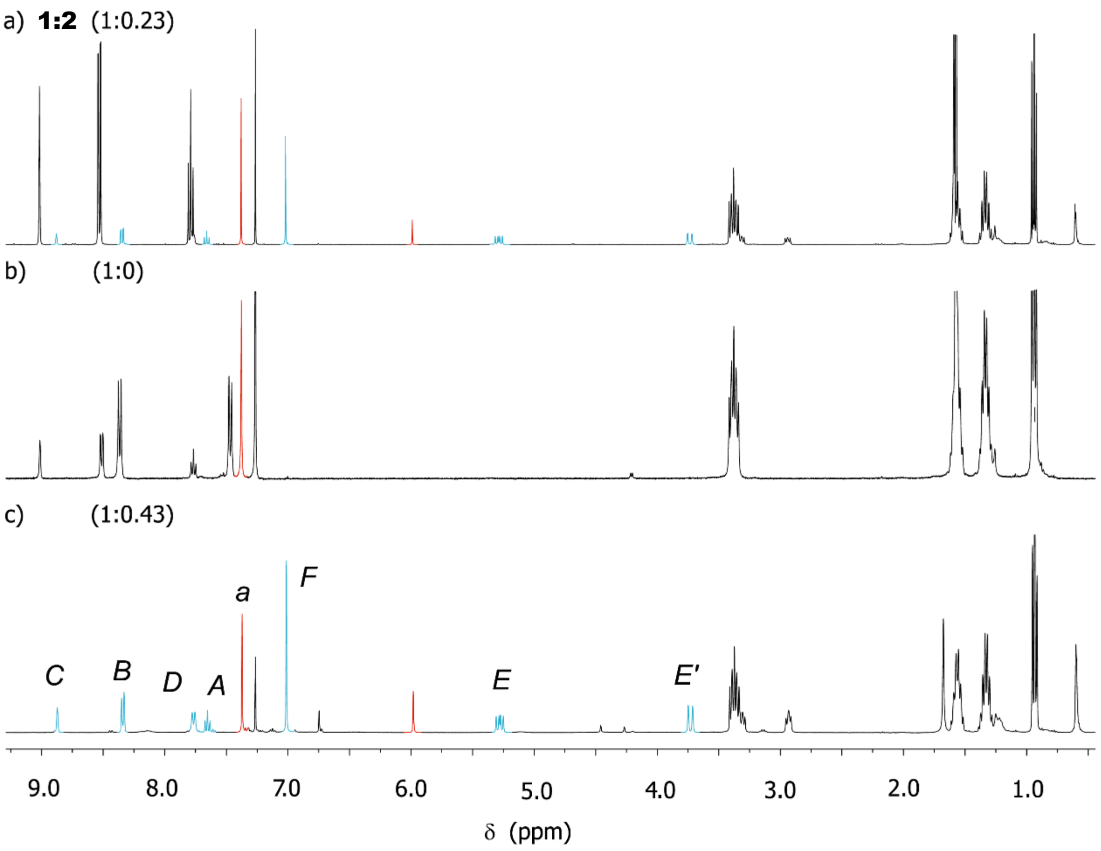

Figure S2. Stack of ^{1}H NMR spectra (400 MHz, $CDCl_3$) of: a) crude reaction with pentafluorophenyl-derived ester **3b**; d) crude reaction with *p*-nitrophenyl-derived ester **3c**; d) crude reaction with *N*-hydroxysuccinimide-derived ester **3d**. Ratios calculated with the integral of the signal related to proton H_a (colored in red) for thread **1** and rotaxane **2**. *All the reactions were carried out in the absence of Et_3N.*

Rotaxane 2

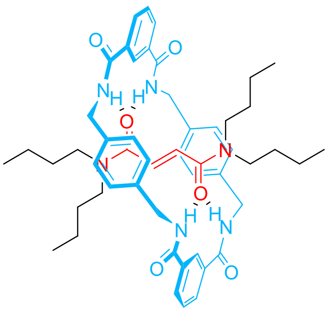

2

Rotaxane **2** was isolated by column chromatography using silica gel as the stationary phase and a mixture of hexane/ethyl acetate (7:1) as the eluent, to give the title product as a white solid. The compound showed identical physical and spectroscopic data as those reported in the literature.[1]

The non-interlocked macrocycle (**Mac**) was also isolated as a byproduct of the reaction.

5. Synthesis of thiourea-based thread 5

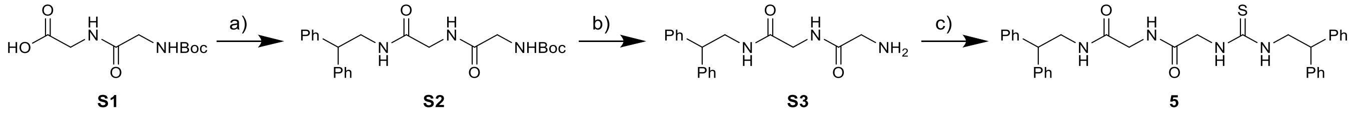

Scheme S2. *Reactions conditions*: a) 2,2-Diphenylethylamine, HOBt, DMAP, EDCI·HCl, CH_2Cl_2, 0 °C to 25 °C, overnight; b) TFA, $CHCl_3$, 0 °C to 25 °C, overnight; c) 2,2-Diphenylethyl isothiocyanate, THF, 25 °C, overnight.

Compound S2

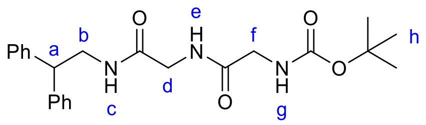

S2

To a stirred solution of *N*-Boc-glycylglycine (**S1**) (2.95 g, 12.7 mmol) in CH_2Cl_2 (100 mL) were added 2,2-diphenylethylamine (3.0 g, 15.2 mmol), HOBt (2.58 g, 19.1 mmol) and DMAP (0.31 g, 2.5 mmol). The solution was cooled at 0 °C and EDCI·HCl (2.97 g, 19.1 mmol) was added. The mixture was stirred at that temperature for 30 min and at room temperature for overnight. After this time, the mixture was washed with HCl 0.1M (2 x 50 mL), NaOH 0.1M (2 x 50 mL) and brine (2 x 50 mL). The organic phase was dried over anhydrous $MgSO_4$, filtered and concentrated to dryness. The resulting crude was suspended in cold hexane and the pure product **S2** was obtained as a white precipitate (3.9 g, 75 %); mp 145-147 °C; **^{1}H-NMR (300 MHz, $CDCl_3$, 298 K) δ**: 7.36 – 7.17 (m, 10H, H_{Ph}), 6.66 (bs, 1H, H_e), 6.07 (bs, 1H, H_c), 5.00 (bs, 1H, H_g), 4.19 (t, J = 8.0 Hz, 1H, H_a), 3.90 (dd, J = 8.0, 5.8 Hz, 2H, H_b), 3.80 (d, J = 5.4 Hz, 2H, H_d), 3.71 (d, J = 5.7 Hz, 2H, H_f), 1.46 (s, 9H, H_h) ppm; **^{13}C-NMR (75 MHz, $CDCl_3$, 298 K) δ**: 170.0 (CO), 168.5 (CO), 156.2 (CO), 141.7 (C), 128.9 (CH), 128.2 (CH), 127.1 (CH), 80.8 (C), 50.6 (CH), 44.4 (CH_2), 43.9 (CH_2), 43.2 (CH_2), 28.5 (CH_3) ppm; **HRMS (ESI)**: calcd for $C_{23}H_{30}N_3O_4$ $[M+H]^+$ 412.2231, found 412.2241; **IR (neat) ν:** 3319.9, 3231.2, 1686.4, 1651.7, 1538.9, 1520.6, 1287.3, 1241.9, 699.1 cm^{-1}.

Compound S3

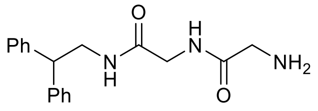

S3

To a cooled at 0 ºC solution of compound **S2** (2.5 g, 6.1 mmol) in $CHCl_3$ (60 mL) was added dropwise TFA (9.4 mL, 121.5 mmol). The mixture was stirred at room temperature for overnight. After this time, the solvent was removed in vacuo and the resulting residue was treated for 2 hours with Amberlyst® basic resin in $CHCl_3$/MeOH (2:1) (40 mL). Then the resin was removed by filtration and the solvent removed under

reduced pressure to obtain the title product **S3** as a white solid, that was employed in the next synthesis step without further purification (1.9 g, 99 %).

Thread 5

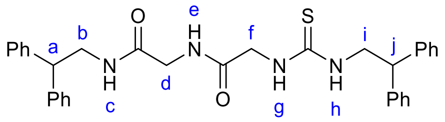

To a stirred biphasic mixture of CH_2Cl_2/THF/$NaHCO_3$ sat. aq. solution (35 mL/3.5 mL/35 mL) was added 2,2-diphenylethylamine (1.33 g, 6.7 mmol). When the amine was completely solved, the mixture was cooled at 0 °C and the stirring was stopped. Then thiophosgene (1.03 mL, 13.4 mmol) was injected into the organic phase. The orange mixture was stirred at 0 °C for 2h. After that, the two phases were separated, and the aqueous phase was extracted with CH_2Cl_2 (2 x 20 mL). The combined organic extracts were dried over anhydrous $MgSO_4$, filtered, and concentrated to dryness to obtain the 2,2-diphenylethyl isothiocyanate as an orange oil. To a stirred solution of this compound in THF (50 mL) was added dropwise a solution of amine **S3** (2.3 g, 7.39 mmol) in THF (50 mL). The mixture was stirred at room temperature for overnight. Then, the solvent was removed under reduced pressure and the crude was dissolved in CH_2Cl_2 (70 mL). The solution was washed with NaOH 1M (2 x 30 mL) and the aqueous phase was extracted again with CH_2Cl_2 (2 x 20 mL). The organic extracts were combined, dried over anhydrous $MgSO_4$, filtered, and concentrated to dryness. The resulting crude was subjected to column chromatography on silica gel using $CHCl_3$/acetone (3:1) as eluent to give the title product as a pale yellow solid (2.59 g, 70%); mp 161-163 °C; **^{1}H-NMR (300 MHz, DMSO-*d*$_6$, 298 K) δ**: 8.22 (s, 1H, H_e), 7.86 (t, J = 5.5 Hz, 1H, H_c), 7.80 (t, J = 5.1 Hz, 1H, H_h), 7.59 (s, 1H, H_g), 7.32 – 7.14 (m, 20H, H_{Ph}), 4.36 (t, J = 7.8 Hz, 1H, H_j), 4.18 (t, J = 7.8 Hz, 1H, H_a), 4.11 – 4.03 (m, 4H, H_{i+f}), 3.73 – 3.66 (m, 2H, H_b), 3.57 (d, J = 5.7 Hz, 2H, H_d) ppm; **^{13}C-NMR (75 MHz, DMSO-*d*$_6$, 298 K) δ**: 182.9 (CS), 169.1 (CO), 168.8 (CO), 142.9 (C), 142.8 (C), 128.6 (CH), 128.5 (CH), 128.0 (CH), 127.9 (CH), 126.5 (CH), 126.4 (CH), 50.1 (CH), 50.0 (CH), 48.3 (CH_2), 47.0 (CH_2), 43.2 (CH_2), 41.9 (CH_2) ppm; **HRMS (ESI)**: calcd for $C_{33}H_{35}N_4O_2S$ $[M+H]^+$ 551.2475, found 551.2487; **IR (neat) ν:** 3291.9, 1645.0, 1518.7, 1493.6, 1218.8, 692.3, 544.8 cm^{-1}.

6. Failed synthesis of thiourea-based rotaxane 6 by using isophthaloyl dichloride (3a)

The synthesis of rotaxane **6** following the general procedure, by employing thiourea-based thread **5** failed due to the rapid degradation of the thread **5**, resulting in a complex mixture of unidentified by-products.

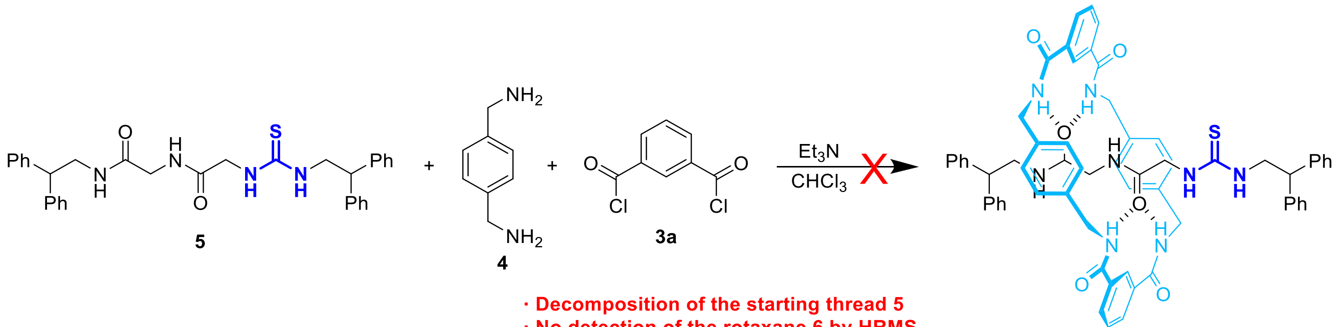

Scheme S3. *Reactions conditions*: isophthaloyl dichloride (**3a**) (8 equiv), *p*-xylylenediamine (**4**) (8 equiv), Et_3N (24 equiv), $CHCl_3$, 25 ºC.

7. Synthesis of thiourea-based rotaxane 6 by using activated esters 3b and 3d as precursors

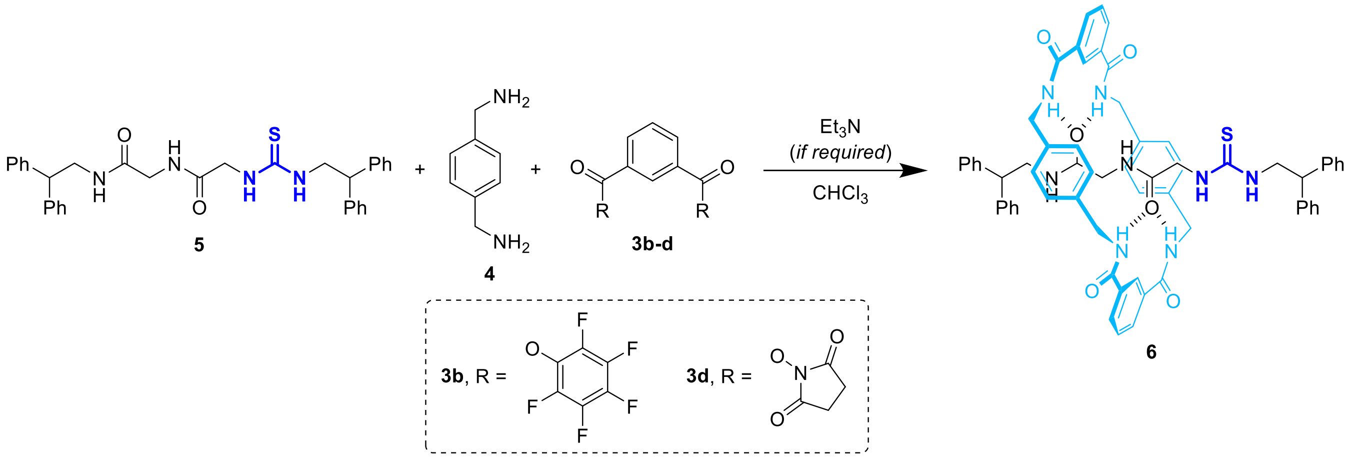

Scheme S4. *Reactions conditions*: isophthaloyl-derived esters (**3b** or **3d**) (8 equiv), *p*-xylylenediamine (**4**) (8 equiv), Et_3N (24 equiv, *if required*), $CHCl_3$, 25 ºC.

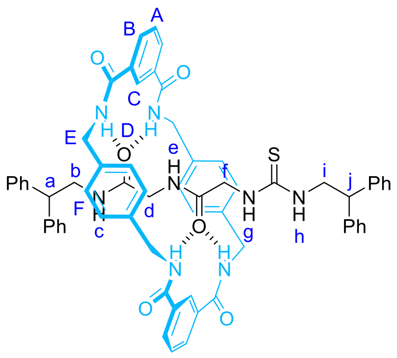

Thiourea-based thread **5** (1 equiv) and Et_3N (24 equiv, *if required*) in anhydrous $CHCl_3$ (500 mL/mmol of thread) were stirred vigorously whilst solutions of *p*-xylylenediamine (**4**) (8 equiv) in anhydrous $CHCl_3$ (20 mL) and the corresponding isophthaloyl derivative **3b** or **3d** (8 equiv) in anhydrous $CHCl_3$ (20 mL) were

simultaneously added (0.06 mL/min) for 5-6 h using motor-driven syringe pumps. After 24 h, the resulting suspension was filtered through a Celite® pad, and the filtrate washed with water (2 x 50 mL), a solution of HCl 1M (2 x 50 mL), a saturated solution of $NaHCO_3$ (2 x 50 mL) and brine (2 x 50 mL). The organic phase was dried over $MgSO_4$ and the solvent removed under reduced pressure. The resulting crude was purified by column chromatography on silica gel using a gradient mixture of $CHCl_3$/MeOH (20:1 to 10:1) as eluent to yield the compound **6** as a white solid (25 % from **3b**; 11 % from **3d**); mp 149-151 °C; **^{1}H-NMR (400 MHz, DMSO-d$_6$, 298 K) δ**: 8.56 (s, 1H, H_C), 8.34 (s, 4H, H_D), 8.07 (dd, *J* = 7.7, 1.4 Hz, 4H, H_B), 7.83 (s, 1H, H_c), 7.78 (s, 1H, H_h), 7.67 (t, *J* = 7.7 Hz, 2H, H_A), 7.29 – 7.10 (m, 18H, H_{Ph+g+e}), 6.94 (s, 8H, H_F), 6.85 (d, *J* = 6.7 Hz, 4H, H_{Ph}), 4.44 (dd, *J* = 13.9, 6.0 Hz, 4H, H_E), 4.36 (t, *J* = 7.7 Hz, 1H, H_j), 4.18 (dd, *J* = 13.8, 3.8 Hz, 4H, $H_{E'}$), 4.07 – 4.02 (m, 2H, H_i), 3.91 – 3.88 (m, 2H, H_f), 3.74 (t, *J* = 7.6 Hz, 1H, H_a), 3.29 – 3.24 (m, 2H, H_b), 2.42 (d, *J* = 3.4 Hz, 2H, H_d) ppm; **^{13}C-NMR (100 MHz, DMSO-d$_6$, 298 K) δ**: 169.3 (CO), 168.1 (CO), 166.0 (CO), 142.7 (C), 142.3 (C), 136.7 (C), 134.3 (C), 130.7 (CH), 128.9 (CH), 128.7 (CH), 128.6 (CH), 128.5 (CH), 128.0 (CH), 127.6 (CH), 126.5 (CH), 126.0 (CH), 49.9 (CH), 49.9 (CH), 48.3 (CH_2), 46.7 (CH_2), 43.5 (CH_2), 43.2 (CH_2), 41.2 (CH_2) ppm (the signal from thiourea carbon is not clearly observed due to its low intensity); **HRMS (ESI)**: calcd for $C_{65}H_{63}N_8O_6S$ $[M+H]^+$ 1083.4586, found 1083.4591; **IR (neat) ν:** 3271.6, 1638.2, 1525.4, 1505.2, 697.1, 599.8, 540.9, 479.2 cm^{-1}.

NOTE:

In the case of the reaction with ester **3b**, Et_3N was added.

In the case of the reaction with ester **3d**, Et_3N was not added.

8. Reaction of thiourea-based thread 5 and rotaxane 6 with substrate 7

To evaluate the reactivity of the thiourea-based thread **5** and rotaxane **6** (nucleophiles) with substrate **7** (electrophile), a mixture of thread **5** or rotaxane **6** (1 equiv) and substrate **7** (2 equiv) was stirred in $CH_2Cl_2$for 48 hours. Upon analysis of the reaction mixtures, it was found that the reaction with thread **5** produced a complex mixture of unidentified products, whereas the reaction with rotaxane **6** yielded compound **9** as the single product.

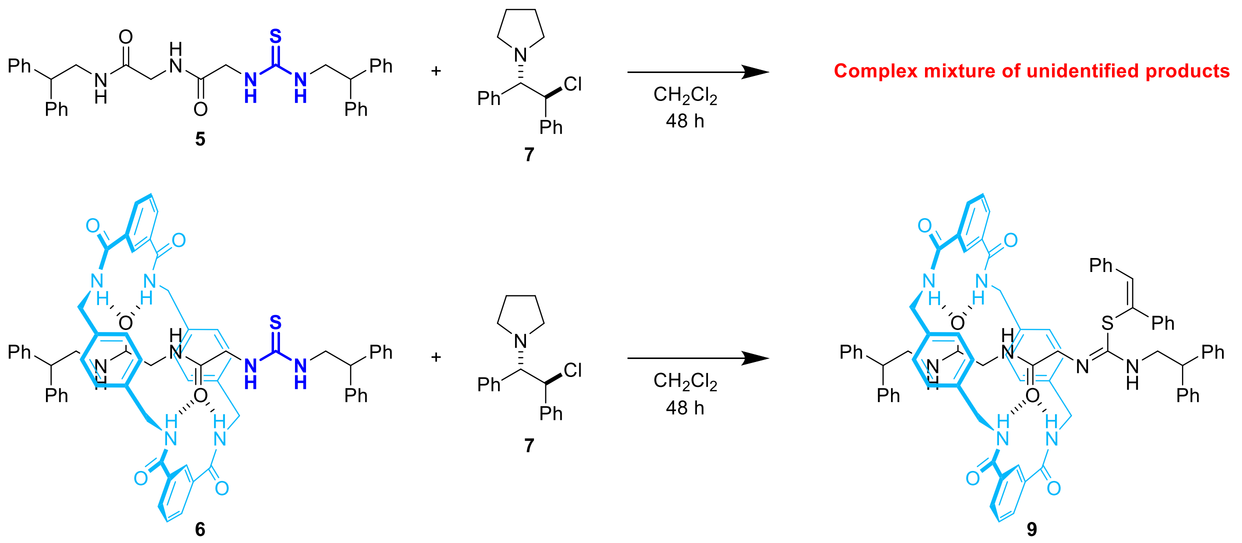

Scheme S5. *Reactions conditions*: thiourea-based thread (**5**) or rotaxane (**6**) (1 equiv), substrate **7** (2 equiv), CH_2Cl_2, 25 ºC, 48 h.

To estimate the disappearance rate of the thiourea-based species, the reactions were followed over time by ^{1}H NMR spectroscopy. The results revealed that the signals associated with thread **5** disappeared completely before 24 hours of reaction, whereas the signals associated with rotaxane **6** remained detectable at this reaction time.

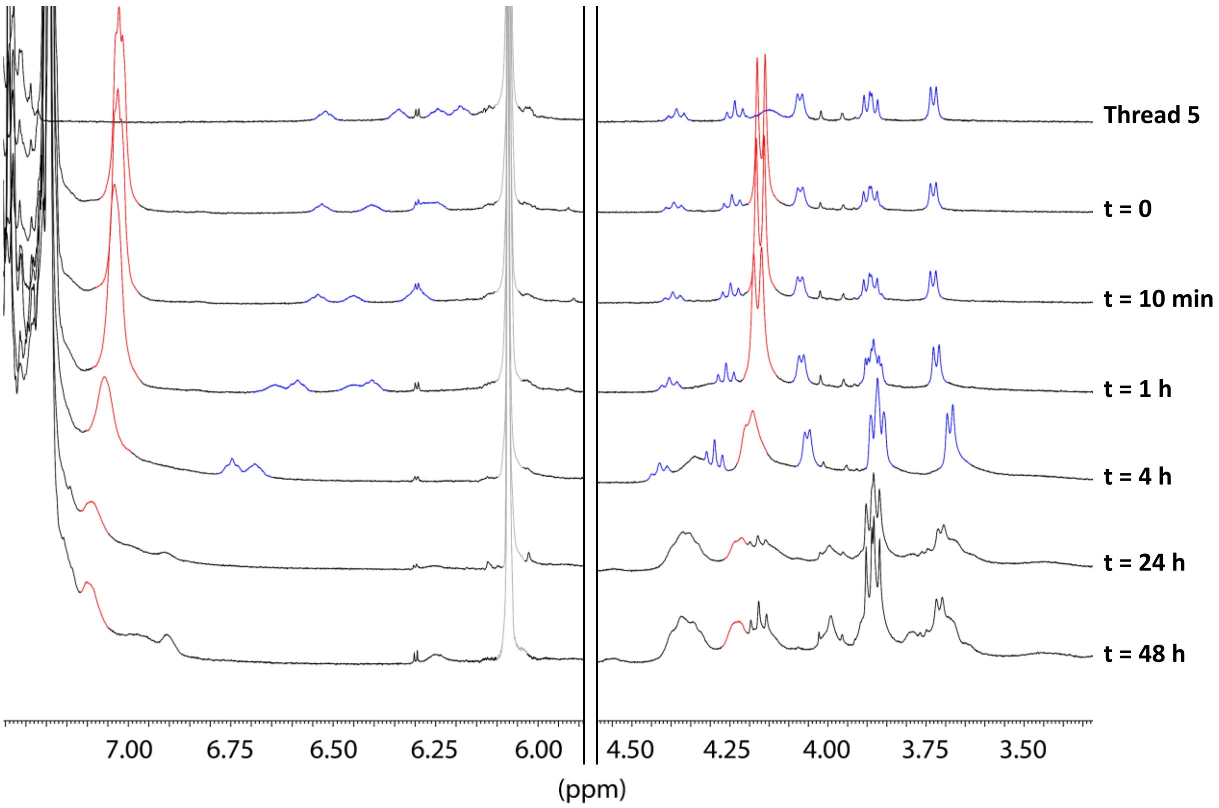

Figure S3. Stacked ^{1}H NMR spectra (400 MHz, CD_2Cl_2, 298 K) of the crude reaction mixture between the thiourea-based thread **5** (blue) and substrate **7** (red) at various reaction times. 1,1,2,2-tetrachloroethane (grey) as internal standard.

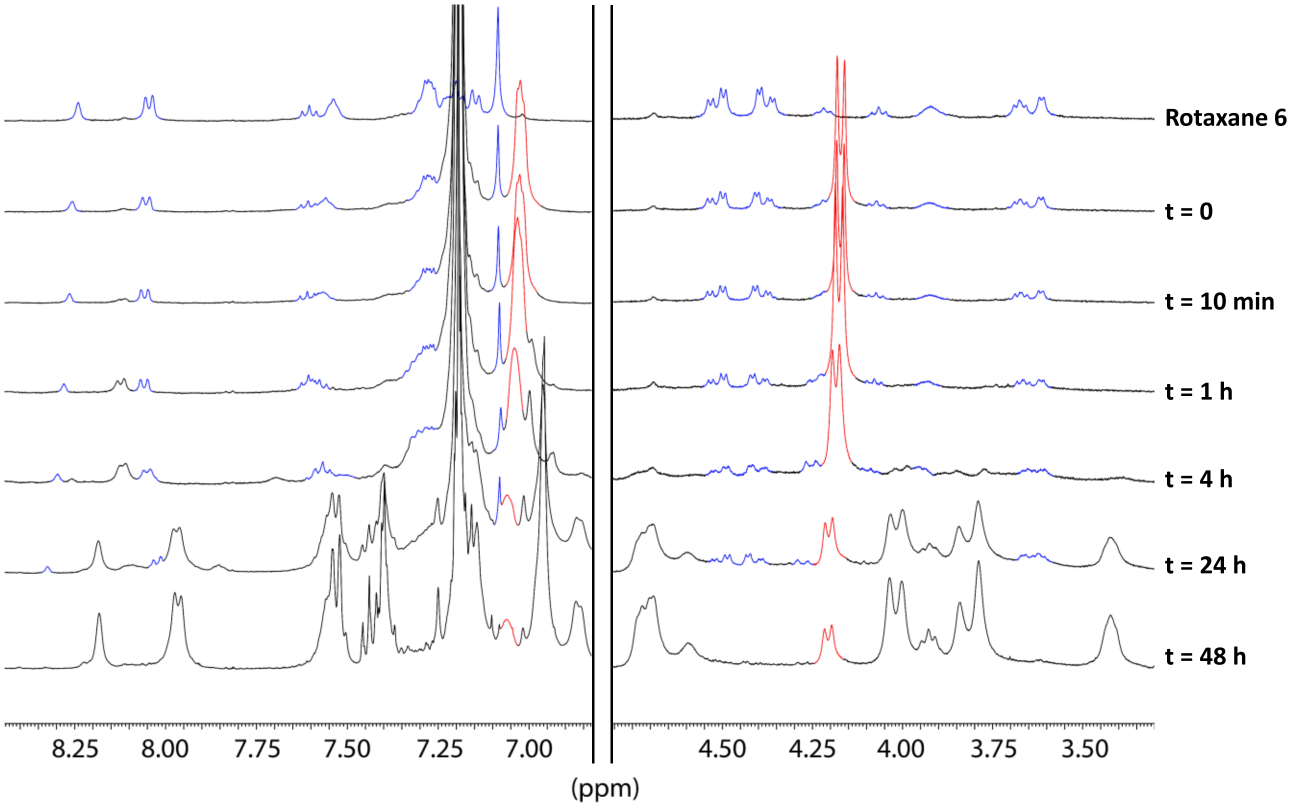

Figure S4. Stacked ^{1}H NMR spectra (400 MHz, CD_2Cl_2, 298 K) of the crude reaction mixture between the thiourea-based rotaxane **6** (blue) and substrate **7** (red) at various reaction times.

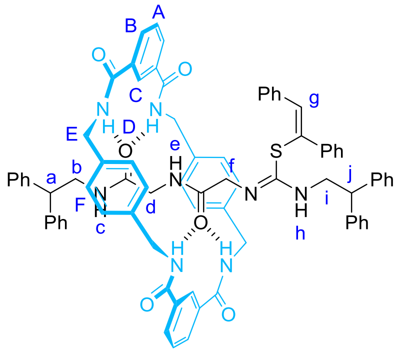

9

To a stirred solution of substrate **7** (15.8 mg, 0.056 mmol) in CH_2Cl_2 (1 mL) was added thiourea-based rotaxane **6** (30 mg, 0.028 mmol). The mixture was stirred for 48 h at room temperature. After this time, the resulting crude was subjected to column chromatography on silica gel using $CHCl_3$/MeOH (30:1) as eluent to give the title product as a white solid (30 mg, 85%); mp 139-141 °C; **^{1}H-NMR (400 MHz, $CDCl_3$, 298 K) δ**: 8.44 (br s, 1H, H_c), 8.03 (s, 2H, H_C), 7.89 (d, J = 7.3 Hz, 4H, H_B), 7.51 – 7.29 (m, 16H, H_{Ph+A+D}), 7.17 (s, 1H, H_g), 7.16 – 7.07 (m, 13H, H_{Ph+e}), 6.96 – 6.90 (m, 12H, H_{Ph+F}), 6.76 (d, J = 7.5 Hz, 4H, H_{Ph}), 4.74 (dd, J = 14.0, 6.4 Hz, 4H, H_E), 4.62 (br s, 1H, H_h), 3.92 (d, J = 13.6 Hz, 4H, $H_{E'}$), 3.86 (t, J = 8.0 Hz, 1H, H_a), 3.81 (br s, 2H, H_f), 3.77 (br s, 3H, H_{i+j}), 3.41 – 3.32 (m, 2H, H_b), 2.57 (br s, 2H, H_d) ppm; **^{13}C-NMR (100 MHz, $CDCl_3$, 298 K) δ**: 172.8 (CO), 169.6 (CO), 166.6 (CO), 150.3 (N=C), 141.5 (CH), 141.2 (C), 141.0 (C), 140.9 (C), 136.7 (C), 135.6 (C), 133.4 (C), 131.4 (CH), 129.6 (CH), 129.3 (CH), 129.0 (CH), 129.0 (CH), 129.0 (CH), 128.9 (CH), 128.9 (CH), 128.5 (CH), 127.8 (CH), 127.6 (CH), 127.3 (CH), 127.2 (CH), 127.2 (CH), 124.7 (CH), 53.2 (CH_2), 50.2 (CH), 50.1 (CH), 46.9 (CH_2), 44.8 (CH_2), 44.5 (CH_2), 41.8 (CH_2) ppm; **HRMS (ESI)**: calcd for $C_{79}H_{73}N_8O_6S$ $[M+H]^+$ 1261.5368, found 1261.5384; **IR (neat) ν:** 3315.0, 1641.1, 1529.3, 1515.8, 699.1, 599.8 cm^{-1}.

9. Phase-transfer nucleophilic fluorination of substrate 7

In a screw-cap vial equipped with a magnetic stirring bar were sequentially added the substrate **7** (0.025 mmol, 1 equiv.), the corresponding catalyst (10 mol%), the CsF (1.2 equiv.) and CD_2Cl_2 (0.1 mL, 0.25 M) as solvent. The vial was sealed, and the reaction mixture was stirred at 1200 rpm at room temperature for 24 h. Aliquots of the crude mixture were analysed by ^{1}H and ^{19}F NMR (4-fluoroanisole as internal standard) to calculate the yield towards fluorinated compound **8**.

Table S1. Evaluation of different catalysts for the phase-transfer fluorination of substrate **7**.[a]

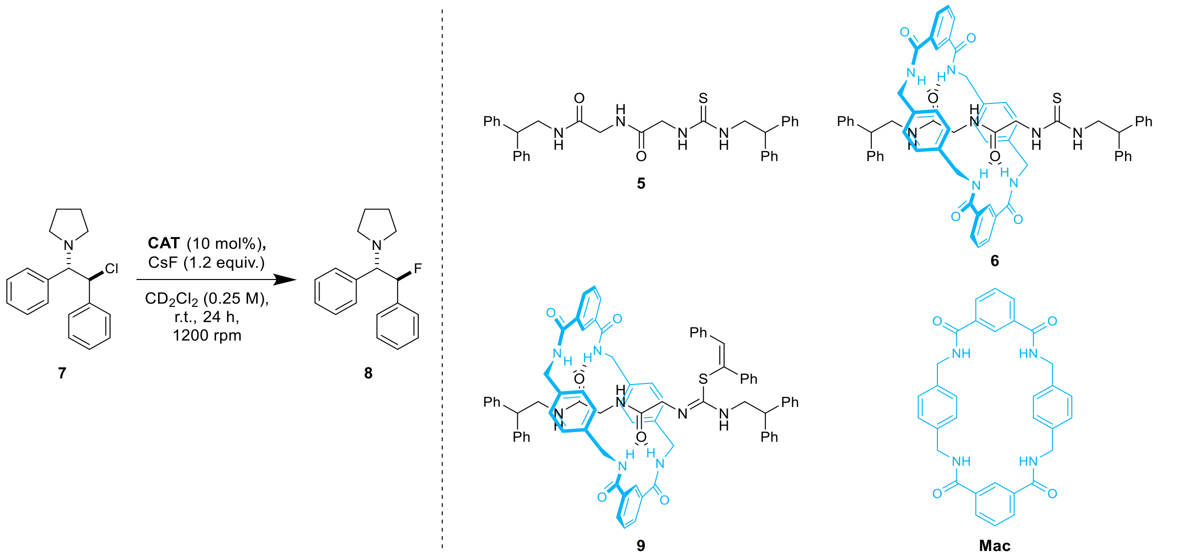

Entry	Catalyst	Yield of 8 (%)[b]	Conversion of 7 (%)[c]
1	--	--	5
2	**5**	8	27
3	**6**	82	91
4	**9**	10	10
5	**Mac**	--	4

[a] **General conditions:** Substrate (0.025 mmol), 0.1 mL of CD_2Cl_2, stirring at 1200 rpm; *CsF was used as provided by the supplier without any prior drying; [b] Determined by ^{19}F NMR using 4-fluoroanisole as internal standard. [c] Determined by ^{1}H NMR of the reaction crudes.

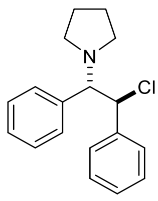

7

Compound **7** was synthesized following a synthetic route described in bibliography and showed identical spectroscopic data as those reported therein[5]; **^{1}H-NMR (300 MHz, $CDCl_3$, 298 K) δ**: 7.19 – 7.08 (m, 8H), 7.00 – 6.95 (m, 2H), 5.43 (d, J = 7.5 Hz, 1H), 4.07 (d, J = 7.5 Hz, 1H), 2.75 – 2.51 (m, 4H), 1.80 – 1.69 (m, 4H) ppm.

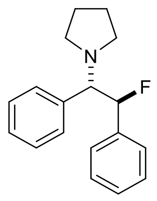

8

Compound **8** was isolated by column chromatography (silica gel) using hexane/Et_2O (9:1 to 8:2 and showed identical spectroscopic data as those reported in bibliography[5]; **^{1}H-NMR (400 MHz, $CDCl_3$, 298 K) δ**: 7.17 – 7.08 (m, 6H), 7.04 – 6.97 (m, 4H), 5.74 (dd, *J* = 46.9, 7.6 Hz, 1H), 3.69 (dd, *J* = 13.2, 7.6 Hz, 1H), 2.81 – 2.68 (m, 2H), 2.61 – 2.53 (m, 2H), 1.82 – 1.72 (m, 4H) ppm; **^{19}F-NMR (376 MHz, $CDCl_3$, 298 K) δ:** -170.86 (dd, *J* = 46.9, 13.2 Hz, 1F) ppm.

10. Stacked ^{1}H NMR spectra of thread 5 and rotaxane 6

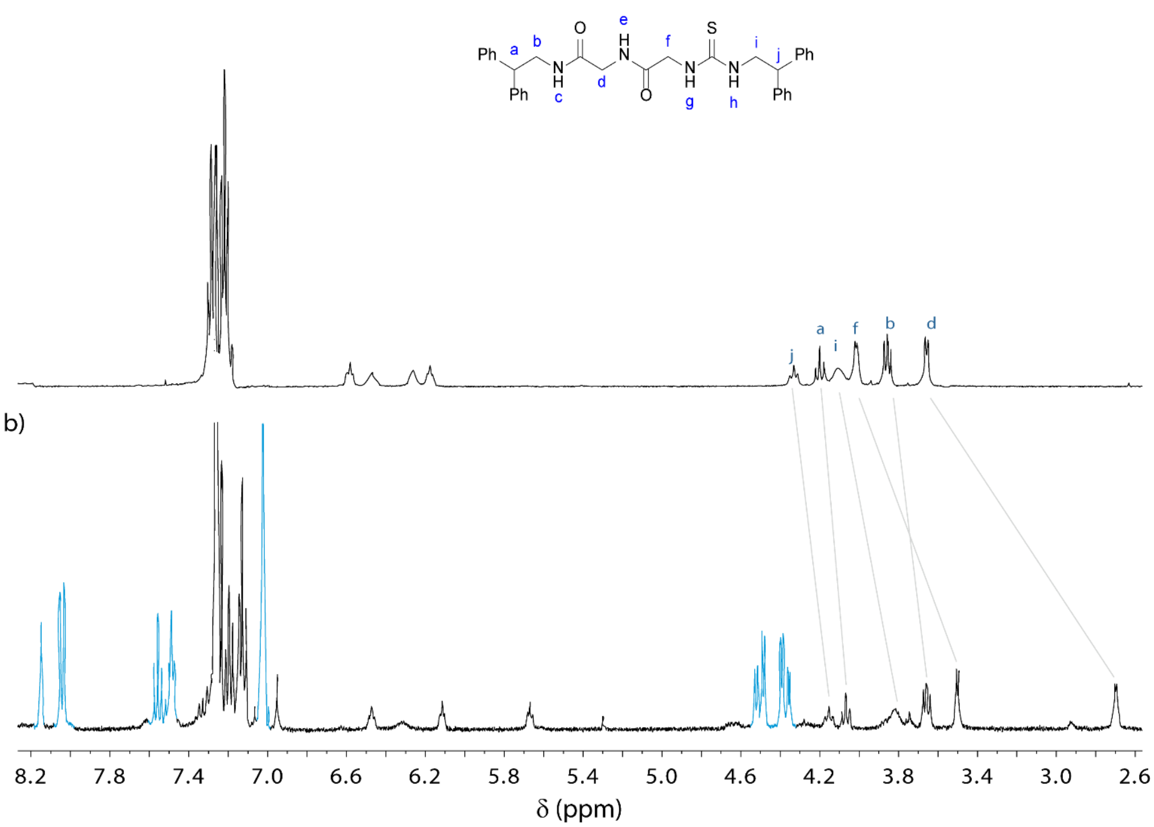

Figure S5. Stacked ^{1}H NMR (400 MHz, $CDCl_3$, 298 K) spectra of: a) thread **5**; b) rotaxane **6**. Signals related with the polyamide macrocycle in **6** are highlighted in light blue.

Table S2. Variation of the chemical shifts of the signals in thread **5** and rotaxane **6**

**Signal**	**δ (ppm)**		**Δ δ (ppm)**
	Thread 5	***Rotaxane 6***	
H_a	4.20	4.07	- 0.13
H_b	3.85	3.66	- 0.19
H_d	3.66	2.70	- 0.96
H_f	4.02	3.50	- 0.51
H_i	4.11	3.82	- 0.29
H_j	4.33	4.15	- 0.18

11. Stacked ^{1}H NMR spectra of rotaxane 6 with increasing amounts of TBAF

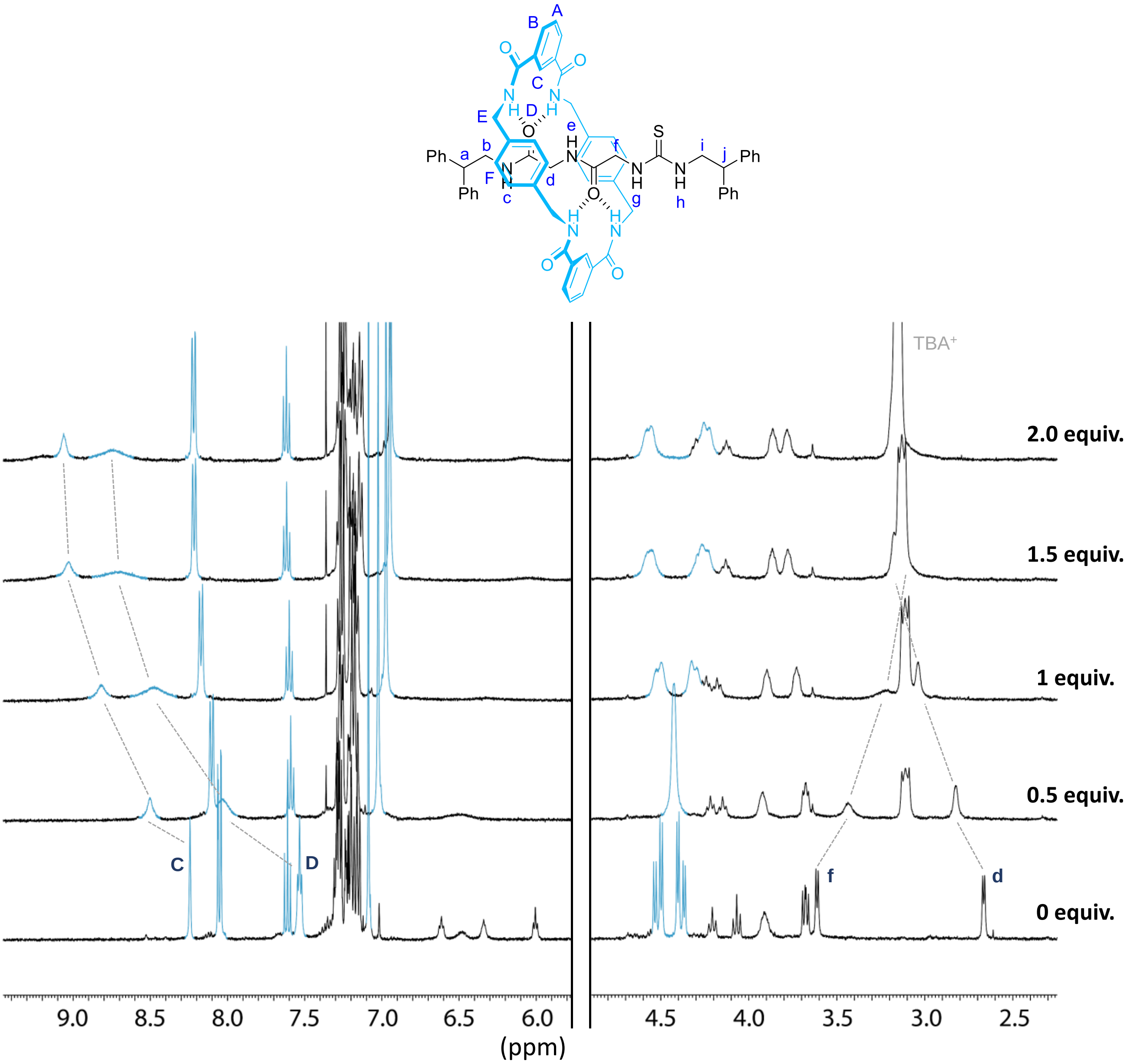

Figure S6. Stacked amplified ^{1}H NMR (400 MHz, CD_2Cl_2, 298 K) spectra of rotaxane **6** at increasing amounts of TBAF·$3H_2O$. Signals related with the polyamide macrocycle are highlighted in light blue.

12. Crystal data and structure refinements for thiourea-based rotaxane 6

Single crystals of $C_{67}H_{67}N_9O_7S$ [JPA_III_79_TBAF_0msp] were obtained by slow cooling of a hot solution of compound **6** in acetonitrile. Intensities were registered at low temperature (100.0 K) on a Bruker D8 QUEST system equipped with a multilayer monochromator and a Mo K/a Incoatec microfocus sealed tube (λ = 0.71073 Å). Absorption corrections were based on multi-scans (program SADABS). Using Olex2,[6] the structure was solved with the SHELXT structure solution program using Intrinsic Phasing and refined with the SHELXL refinement package using Least Squares minimisation[7,8] Hydrogen atoms were included using a riding model. One molecule of water and one molecule of acetonitrile is included in the asymmetric unit. The structure was deposited with CSD (deposition number CCDC-2415530).

Table S3. Crystal data and structure refinement for **6**.

Empirical formula	$C_{67}H_{67}N_9O_7S$	
Formula weight	1142.35	
Temperature	100 K	
Wavelength	0.71073 Å	
Crystal system	Monoclinic	
Space group	C2/c	
Unit cell dimensions	a = 38.173(6) Å	α = 90 °
	b = 11.4040(16) Å	β = 121.999(4) °
	c = 32.413(4) Å	γ = 90 °
Volume	11966(3) $Å^3$	
Z	8	
Density (calculated)	1.268 g/cm^3	
Absorption coefficient	0.117 mm^{-1}	
F(000)	4832.0	
Crystal size	0.24 x 0.08 x 0.06 mm^3	
Theta range for data collection	3.786 to 61.168 °	
Index ranges	$-54<h<54$, $-16<k<16$, $-45<l<46$	
Reflections collected	224157	
Independent reflections	18315 [R_{int}= 0.0778]	
Absorption correction	Semi-empirical from equivalents	
Refinement method	Full-matrix least-squares on F2	
Data / restraints / parameters	18315/0/761	
Goodness-of-fit on F2	1.161	
Final R indices [I>2sigma(I)]	R_1= 0.0565, wR_2= 0.1250	

R indices (all data)	R_1= 0.1100, wR_2= 0.1711
Largest diff. peak and hole	0.55/-0.40 e Å^{-3}

Table S4. Hydrogens bonds for **6** [Å and (°)]

D-H...A	d(D-H)	d(H...A)	d(D...A)	<(DHA)
O1SH1SD…O3[1]	0.87	1.88	2.746(2)	177.7
O1SH1SE…O4[2]	0.87	1.97	2.820(2)	166.8
N1H1…O5[3]	0.88	2.07	2.905(2)	157.9
N2H2…O5[3]	0.88	2.22	3.028(2)	151.7
N3H3…O1S	0.88	2.01	2.859(2)	162.9
N4H4…O2	0.88	1.98	2.843(2)	167.6
N5H5…O2	0.88	2.22	3.081(2)	167.3
N6H6…O2	0.88	2.22	3.100(2)	175.1
N7H7…O1	0.88	2.23	2.968(2)	141.3
N8H8…O1[4]	0.88	2.23	3.009(2)	146.7

Symmetry transformations used to generate equivalent atoms:

[1] 1/2-X,-1/2+Y,1/2-Z; [2] 1/2-X,1/2+Y,1/2-Z; [3] 1/2-X,1/2-Y,1-Z; [4] 1/2-X,3/2-Y,1-Z

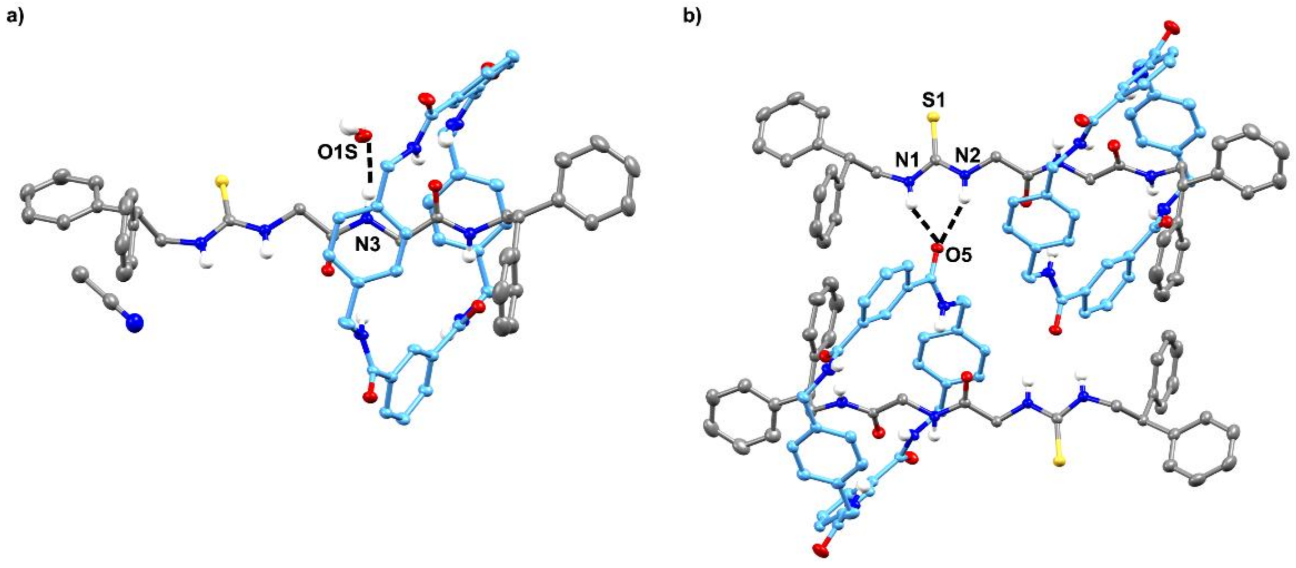

Figure S7. Molecular structure of rotaxane **6** with thermal ellipsoids drawn at 50% probability, showing the intermolecular hydrogen-bonding interactions: a) of rotaxane **6** with a water molecule; b) between two molecules of rotaxane **6**. For clarity, selected hydrogens atoms have been deleted.

13. ^{1}H, ^{13}C and ^{19}F NMR Spectra of synthesized compounds

3b (^{1}H NMR, 300 MHz, $CDCl_3$, 298 K)

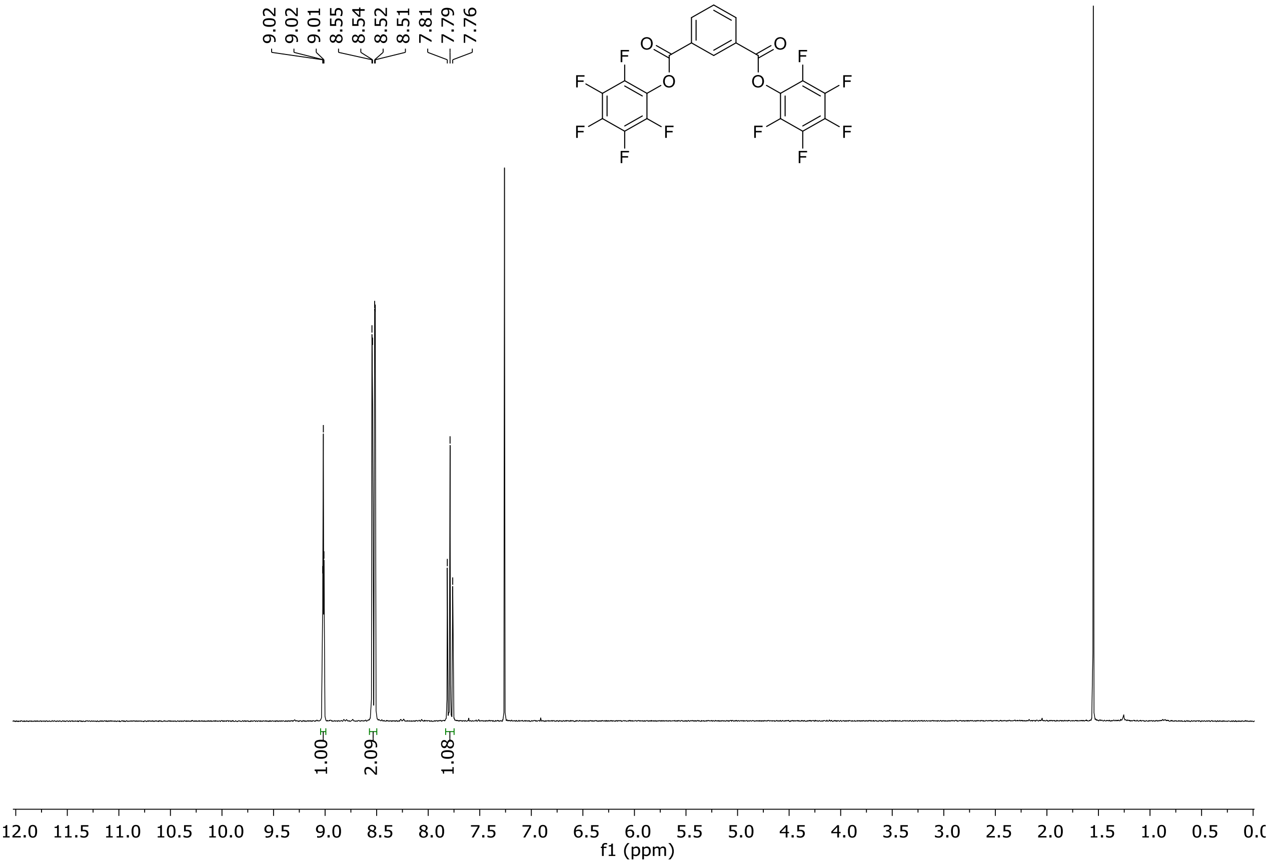

3b (^{19}F NMR, 282 MHz, $CDCl_3$, 298 K)

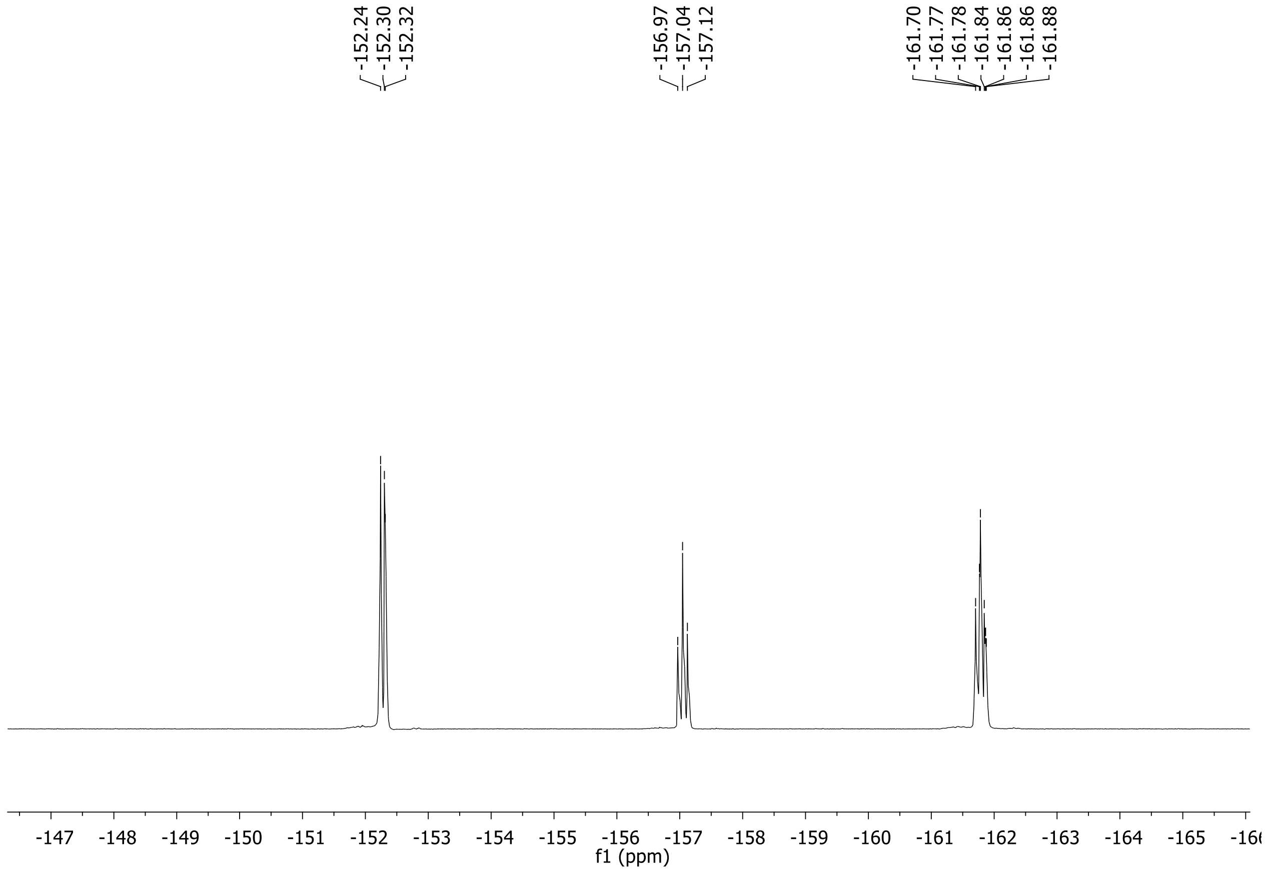

3c (^{1}H NMR, 300 MHz, DMSO-d_6, 345K)

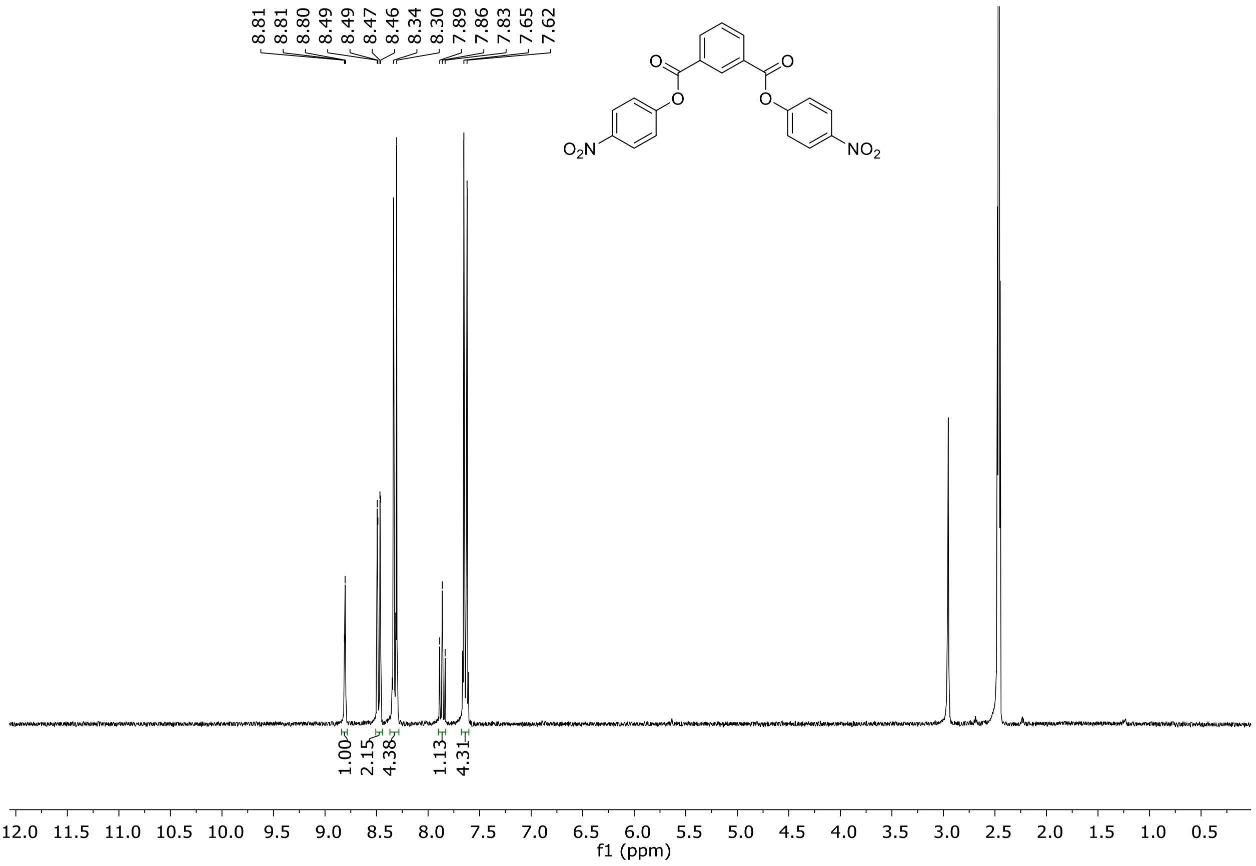

3d (^{1}H NMR, 300 MHz, $CDCl_3$, 298 K)

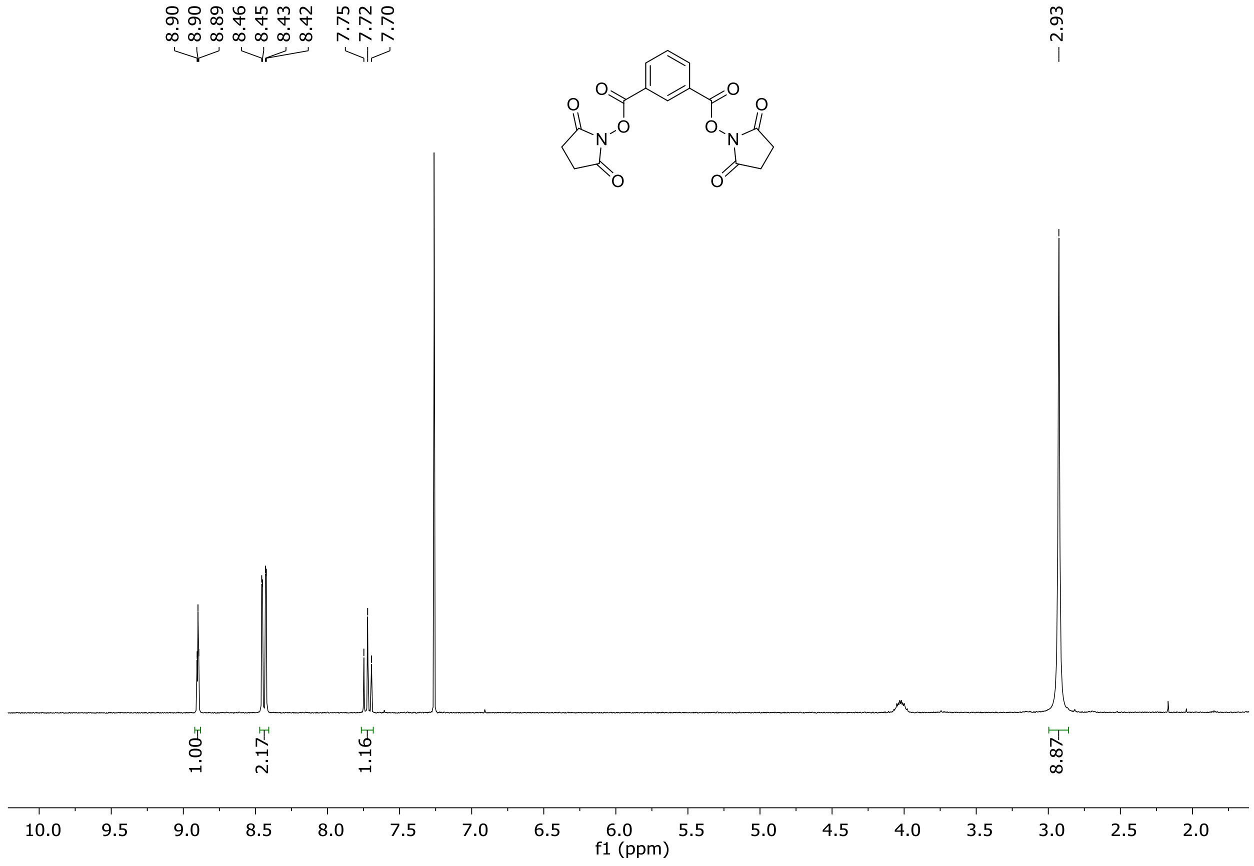

S2 (^{1}H NMR, 300 MHz, $CDCl_3$, 298 K)

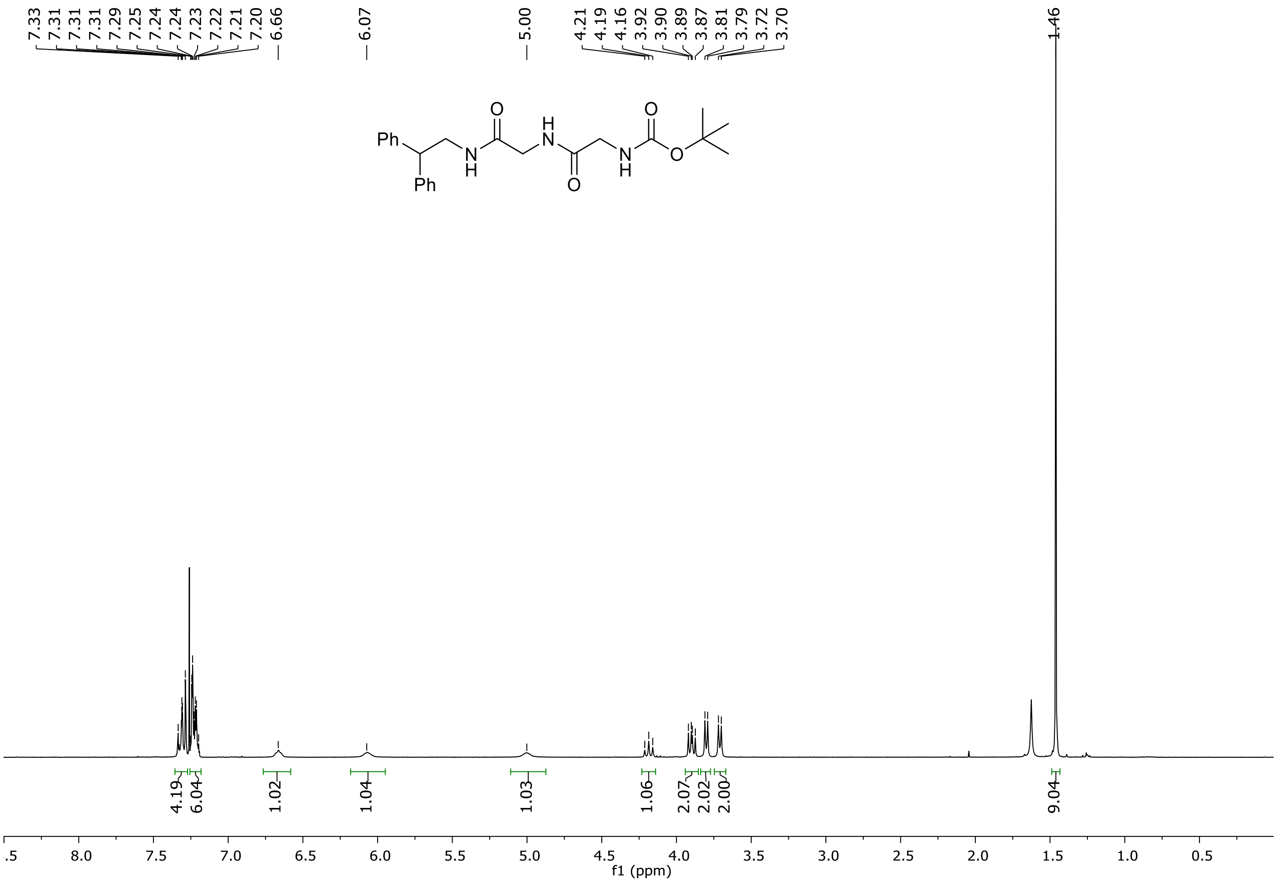

S2 (COSY ^{1}H-^{1}H NMR, 300 MHz, $CDCl_3$, 298 K)

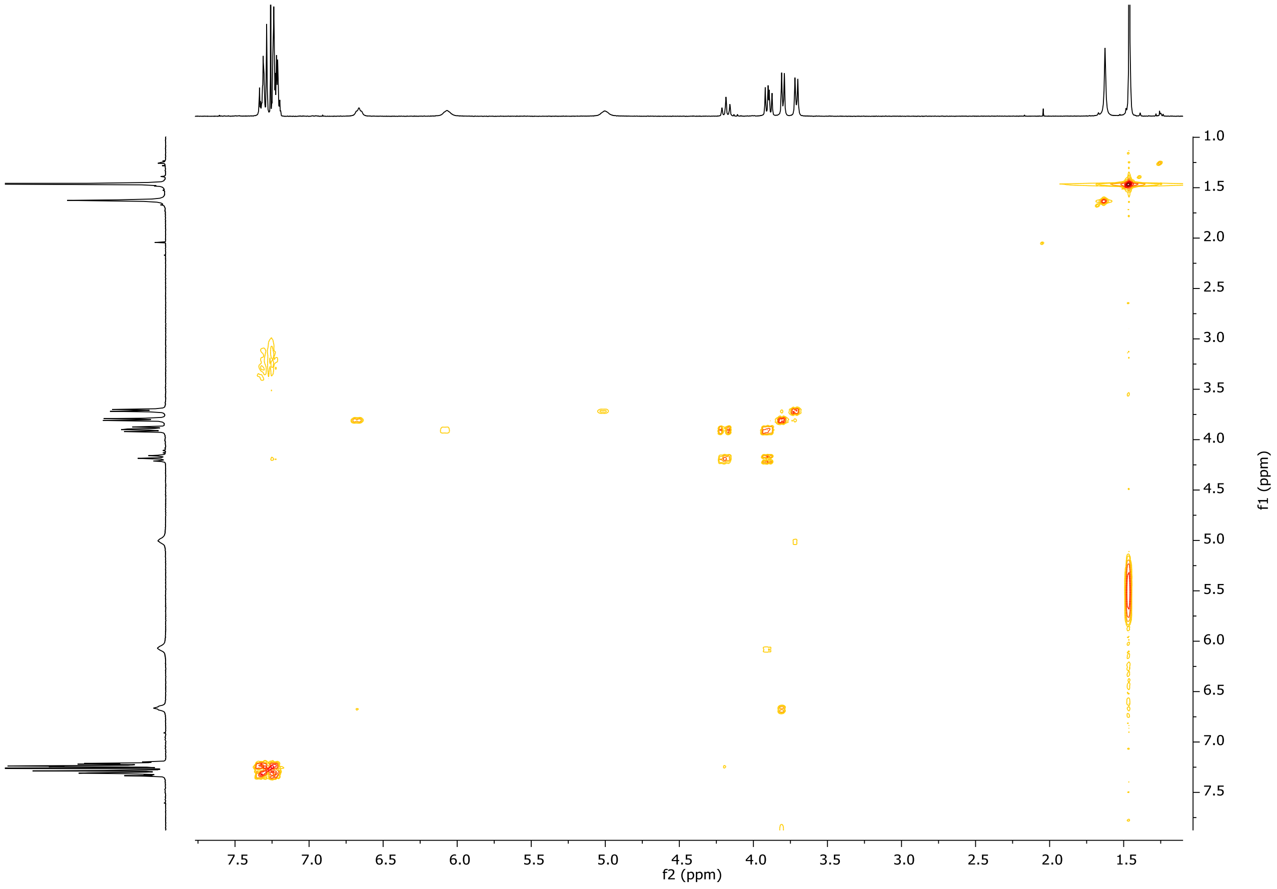

S2 (^{13}C NMR, 75 MHz, $CDCl_3$, 298 K)

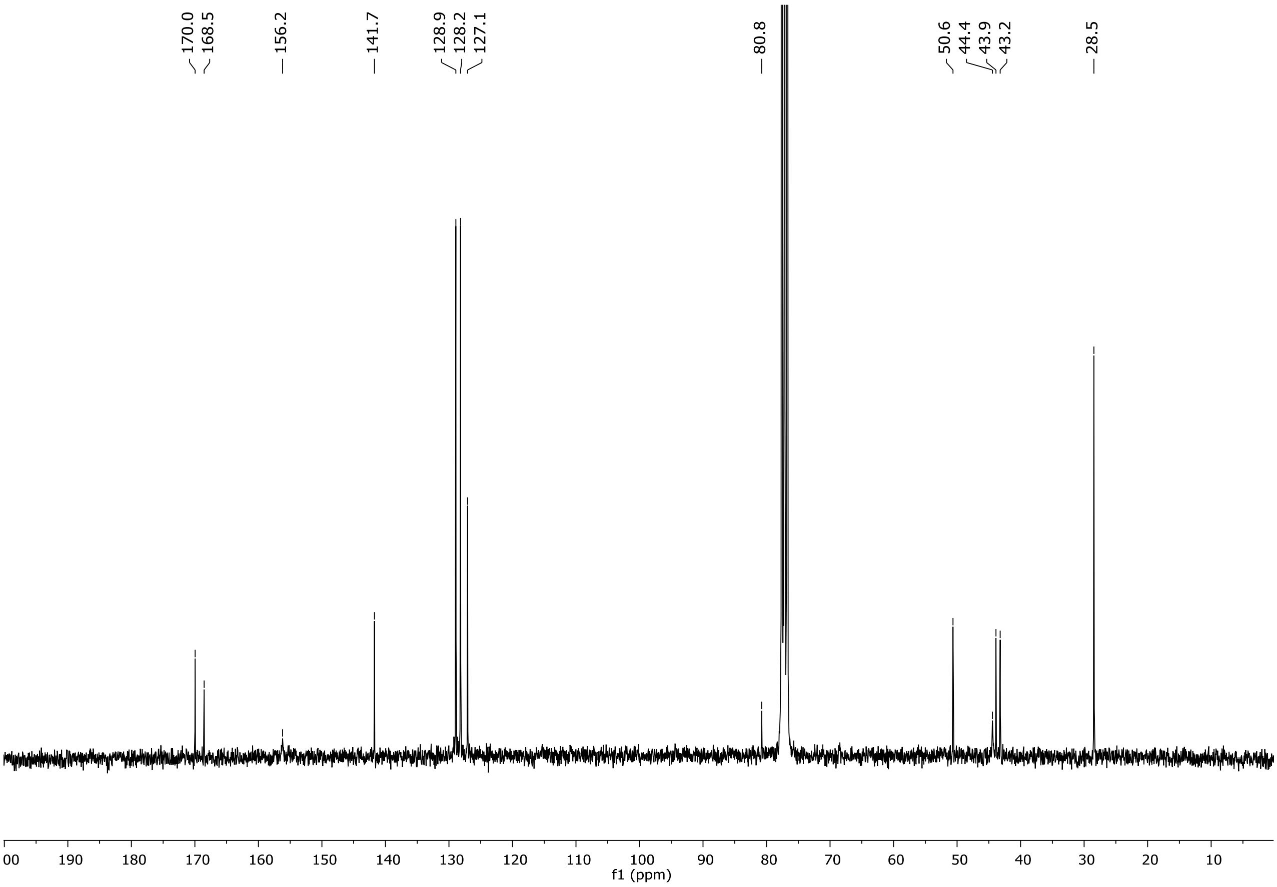

S2 (DEPT ^{13}C NMR, 75 MHz, $CDCl_3$, 298 K)

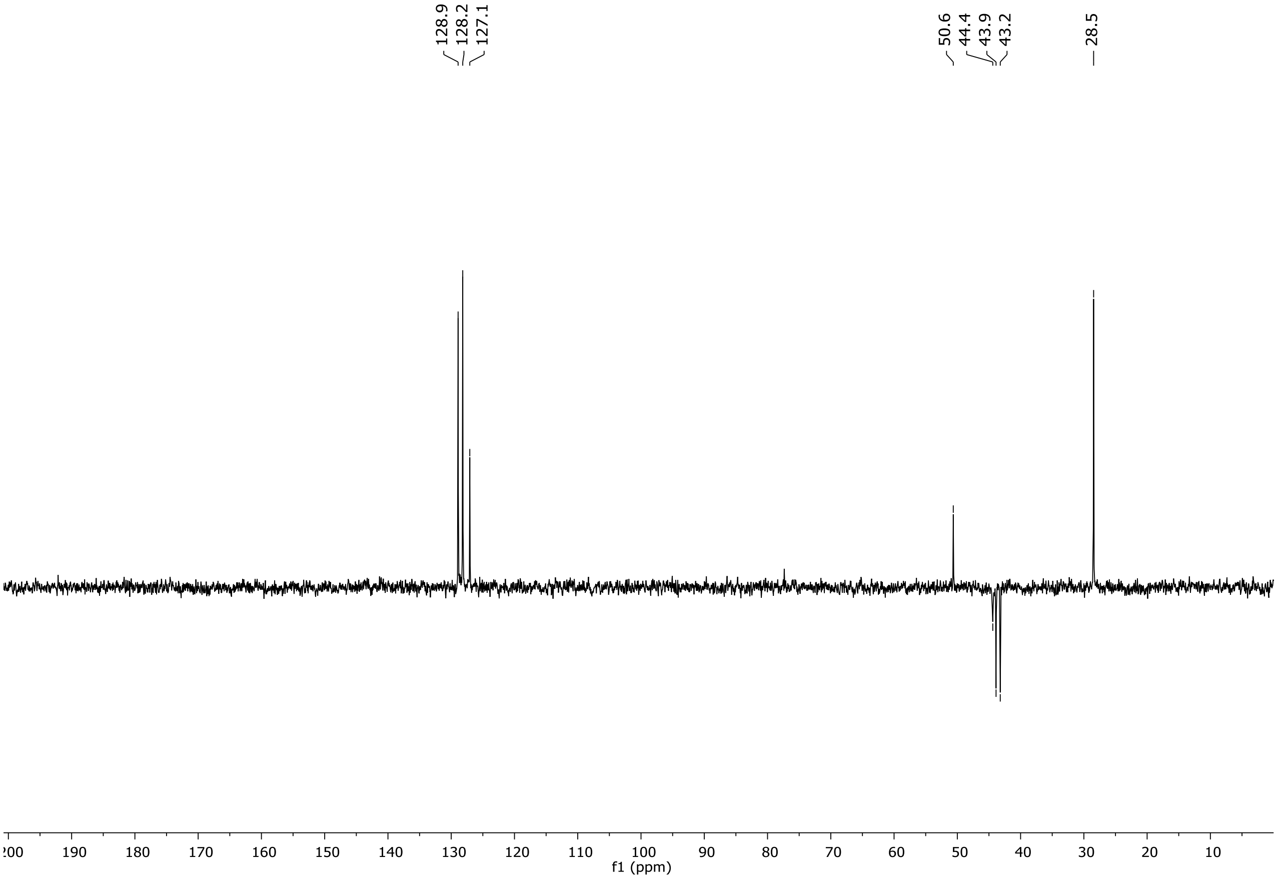

5 (^{1}H NMR, 300 MHz, DMSO-d$_6$, 298 K)

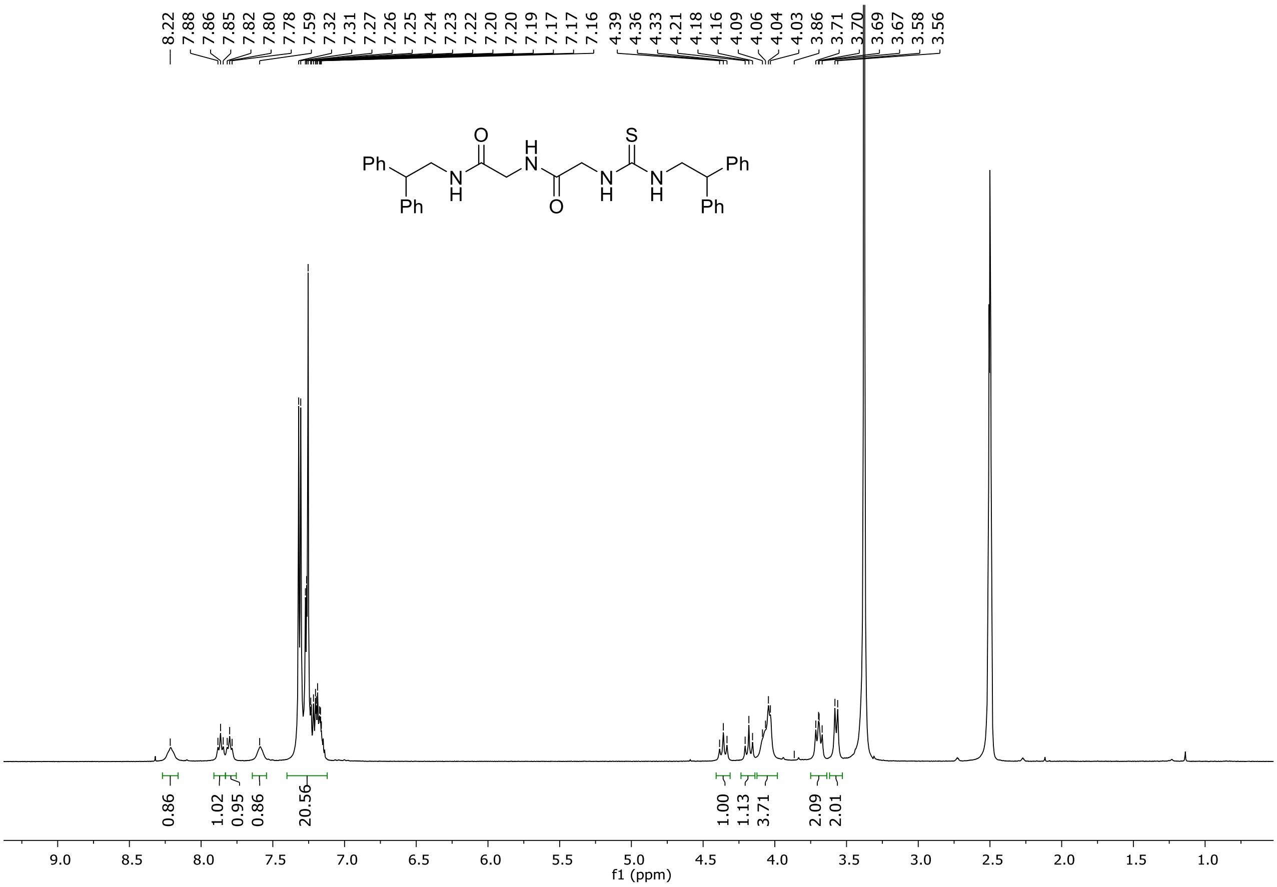

5 (COSY ^{1}H-^{1}H NMR, 300 MHz, $CDCl_3$, 298 K)

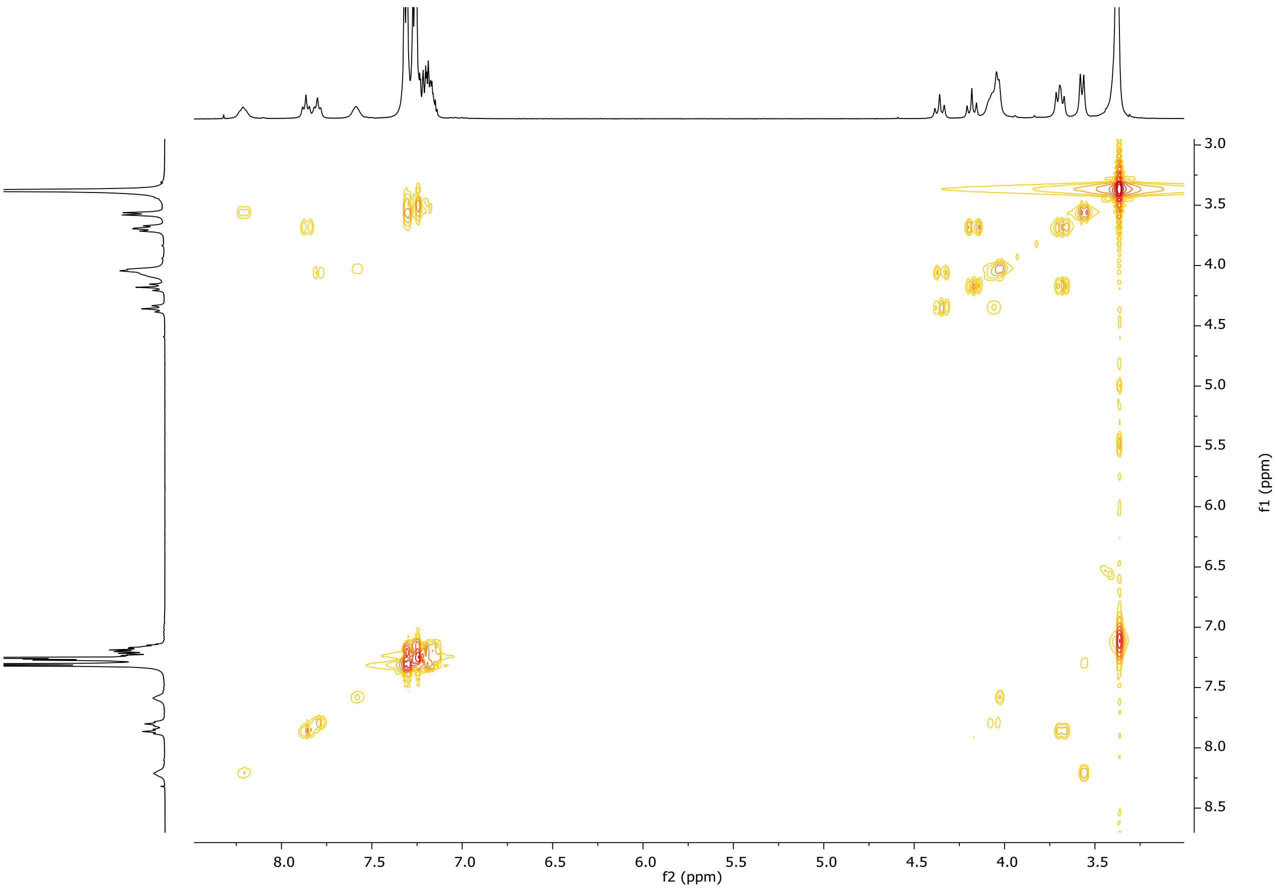

5 (^{13}C NMR, 75 MHz, DMSO-d_6, 298 K)

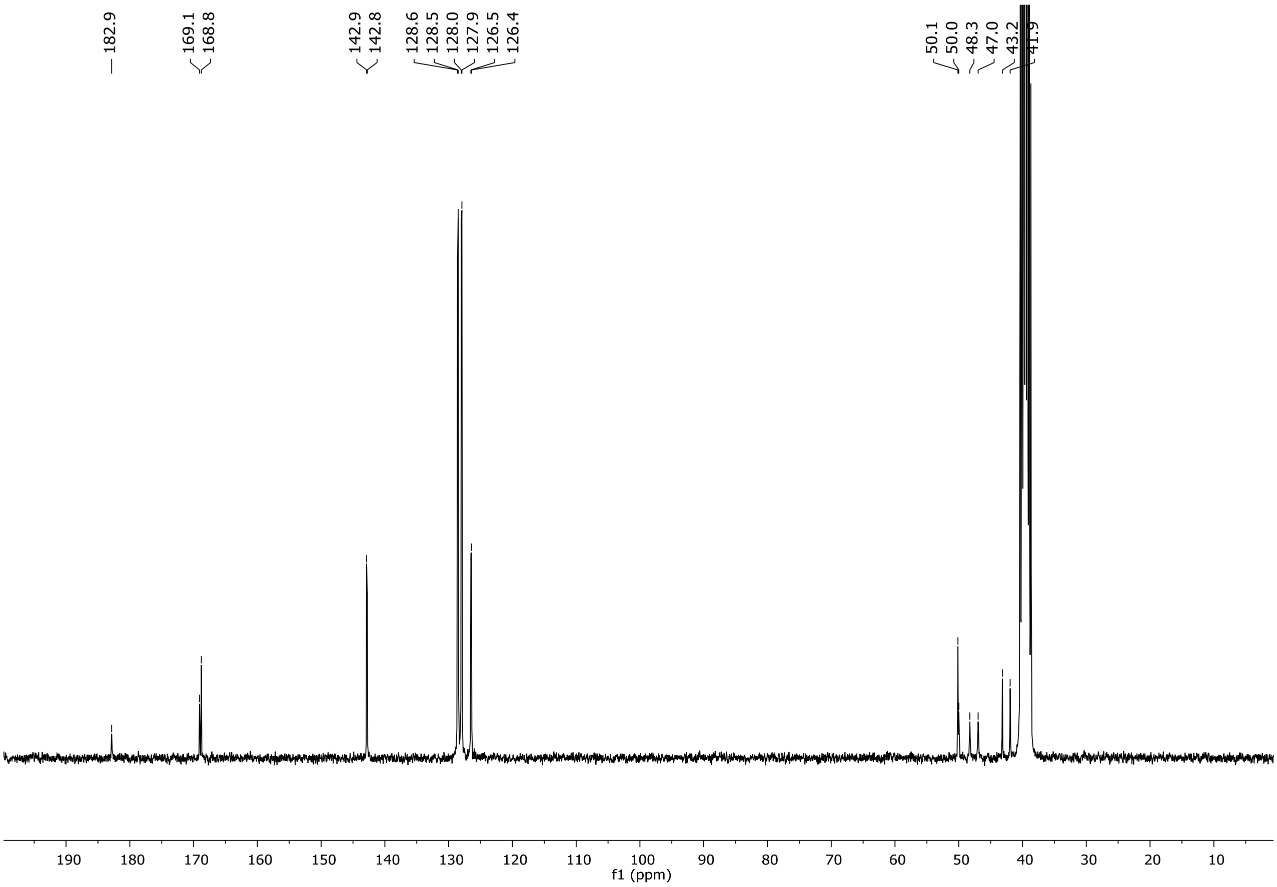

5 (DEPT ^{13}C NMR, 75 MHz, DMSO-d_6, 298 K)

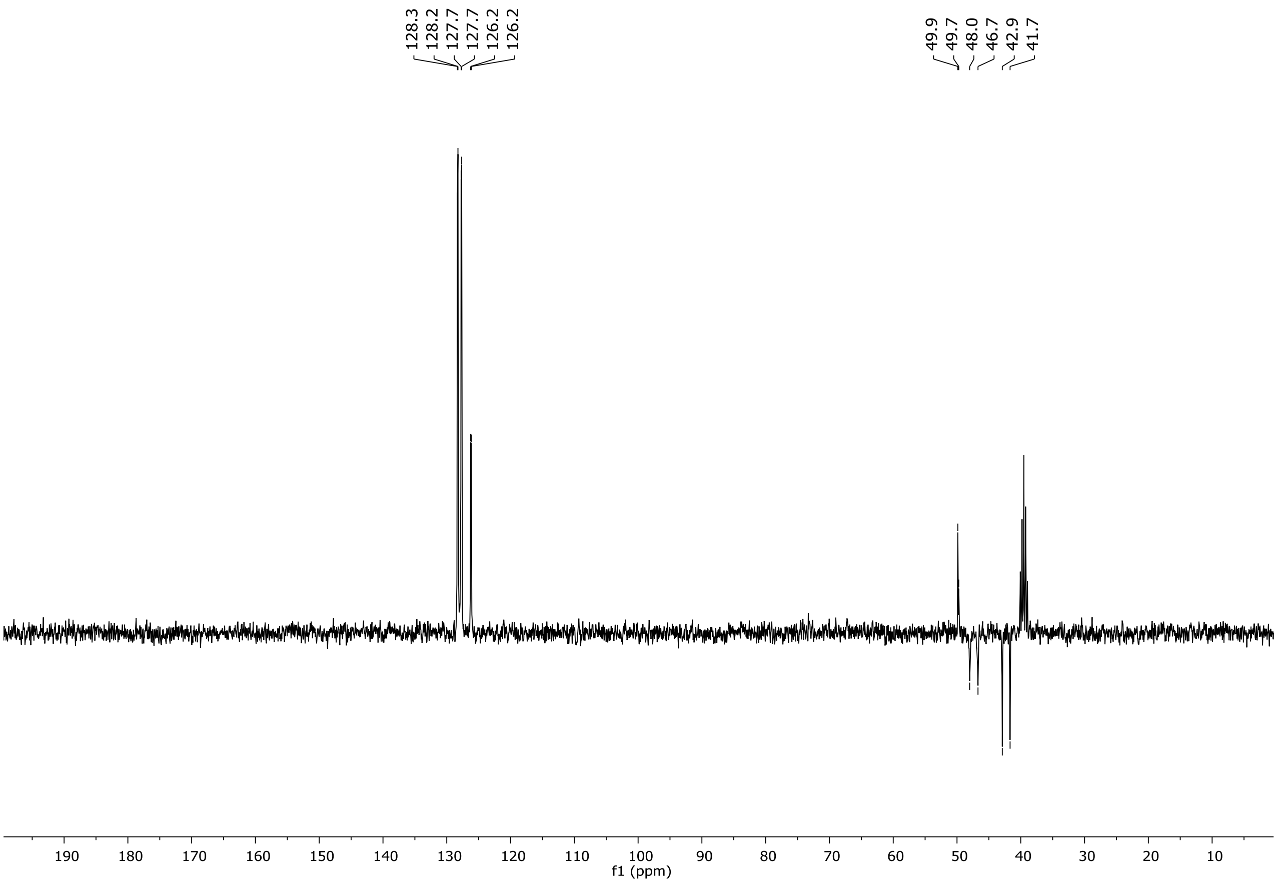

5 (HMBC ^{1}H-^{13}C NMR, 300 MHz, DMSO-d_6, 298 K)

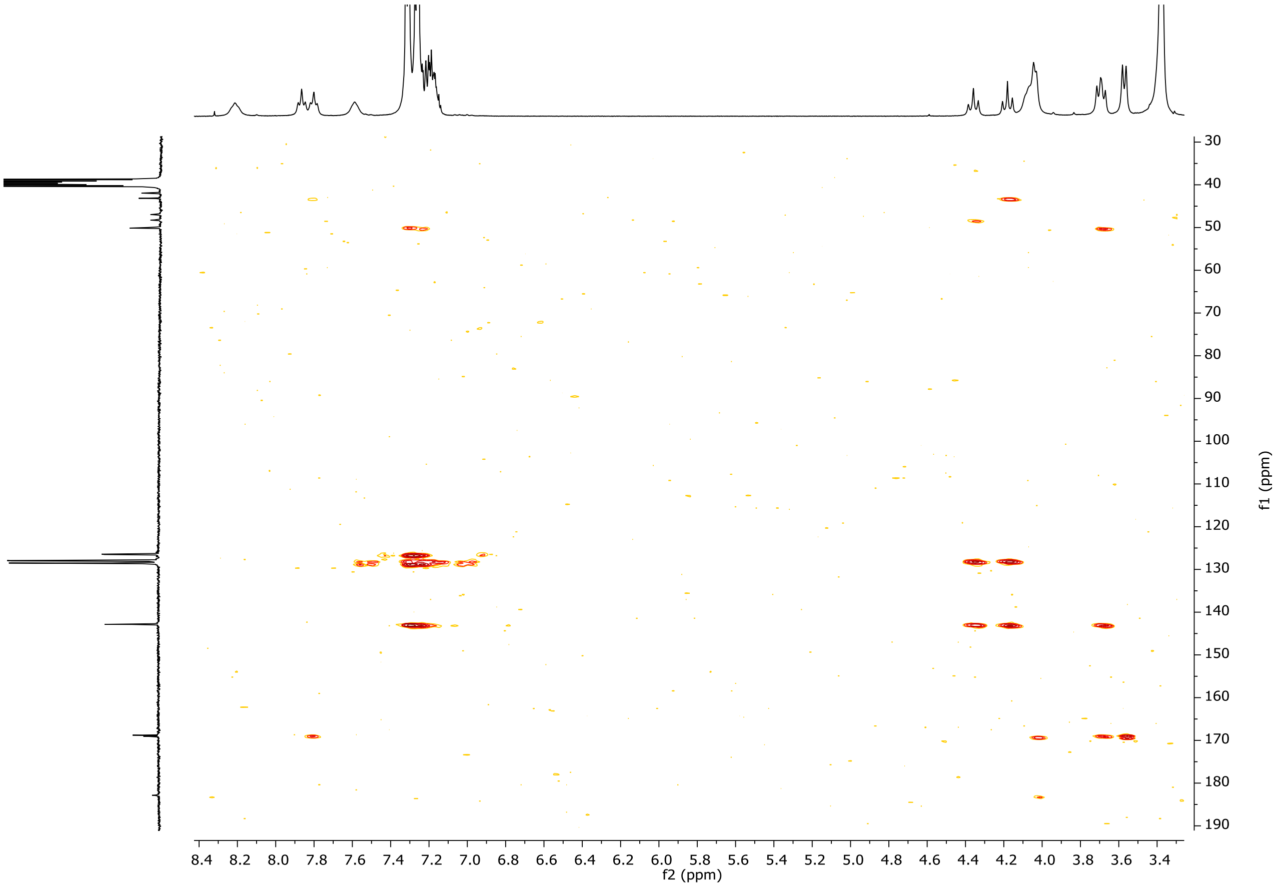

6 (^{1}H NMR, 400 MHz, DMSO-d_6, 298K)

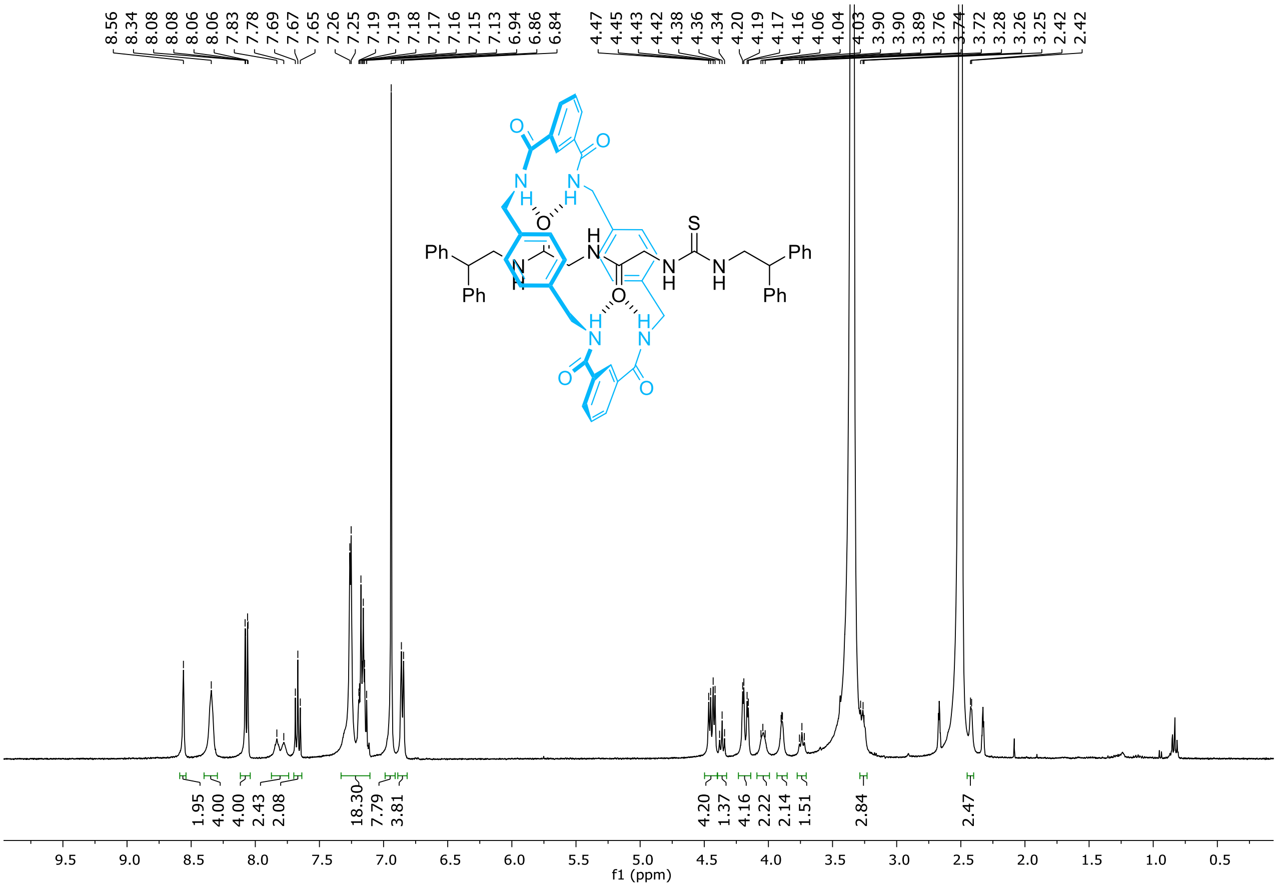

6 (COSY ^{1}H-^{1}H NMR, 400 MHz, DMSO-d_6, 298K)

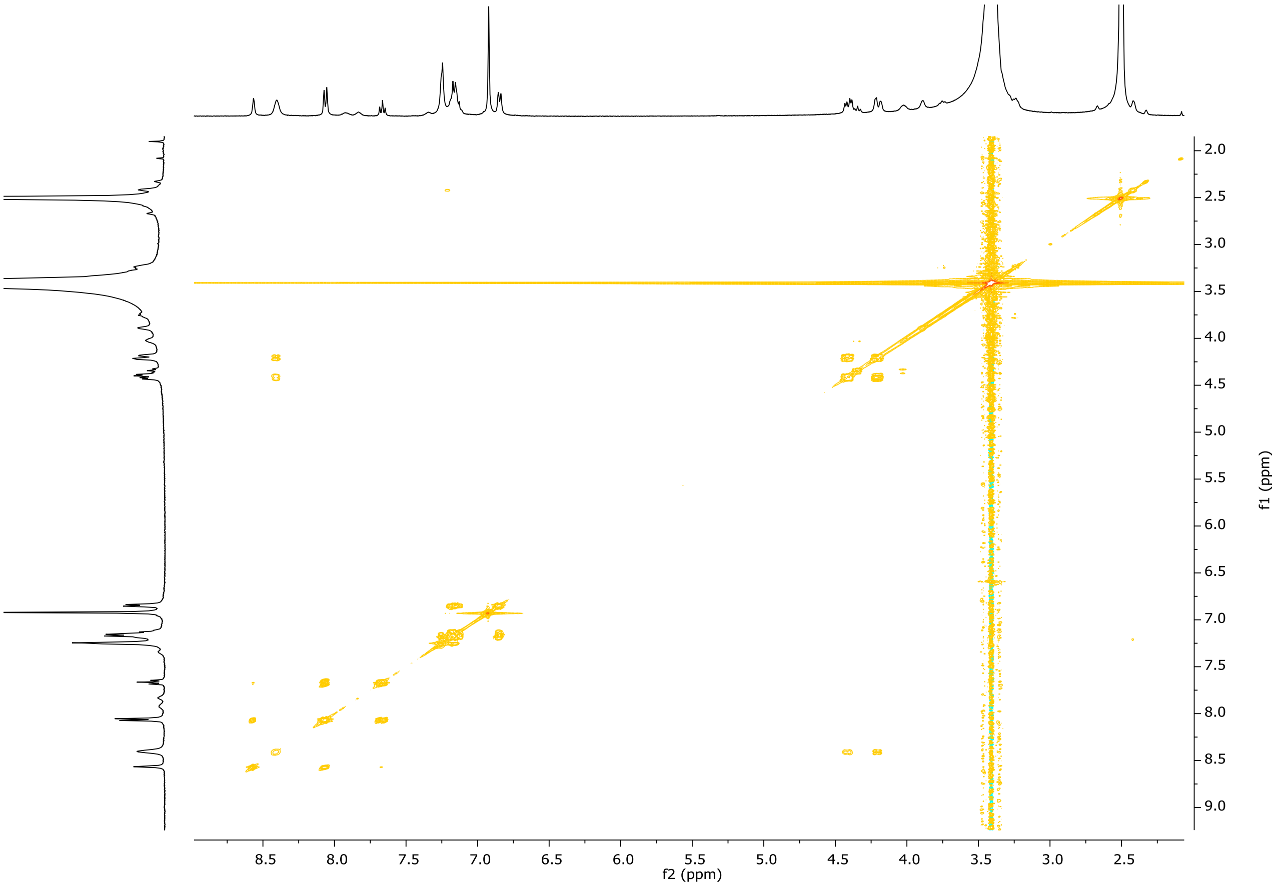

6 (^{13}C NMR, 400 MHz, DMSO-d_6, 298K)

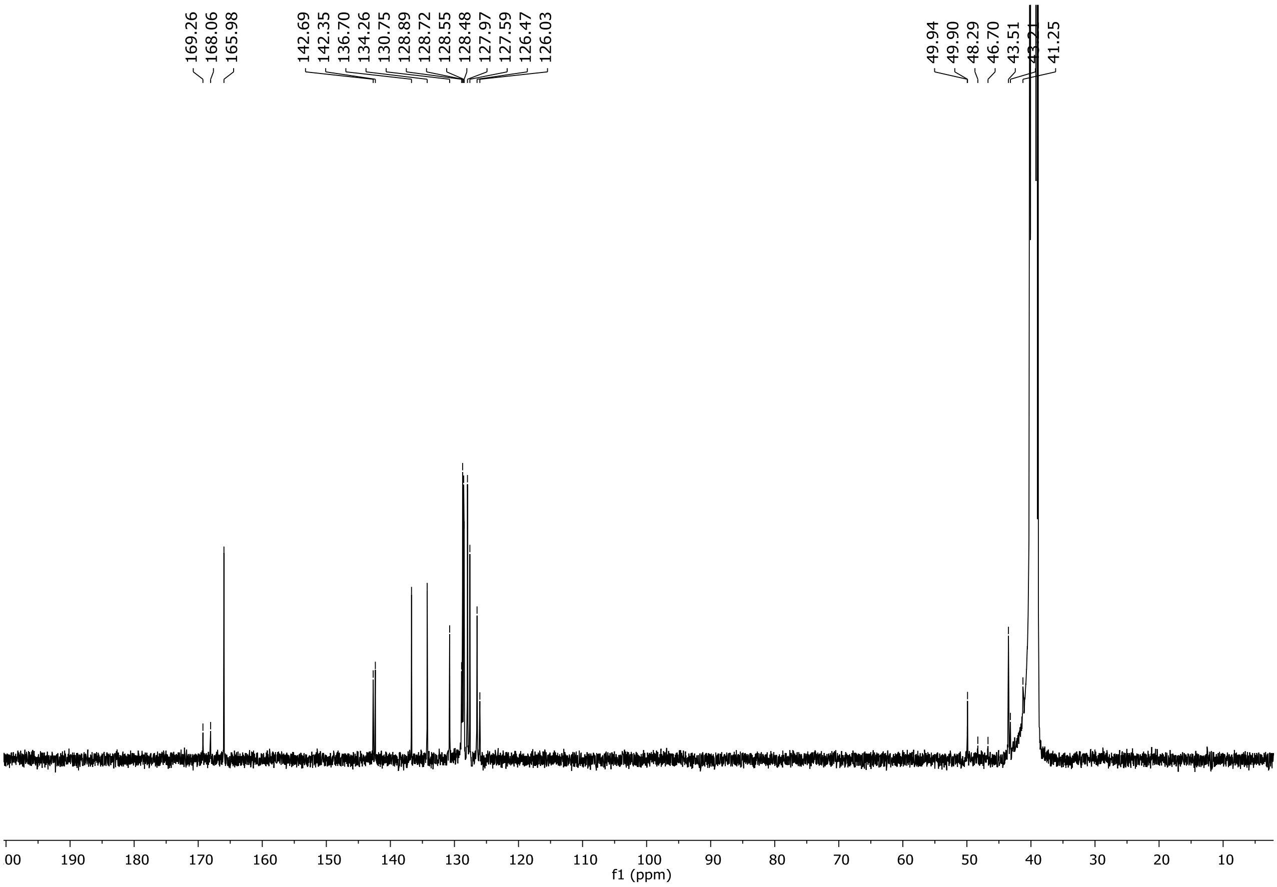

6 (DEPT ^{13}C NMR, 400 MHz, DMSO-d$_6$, 298K)

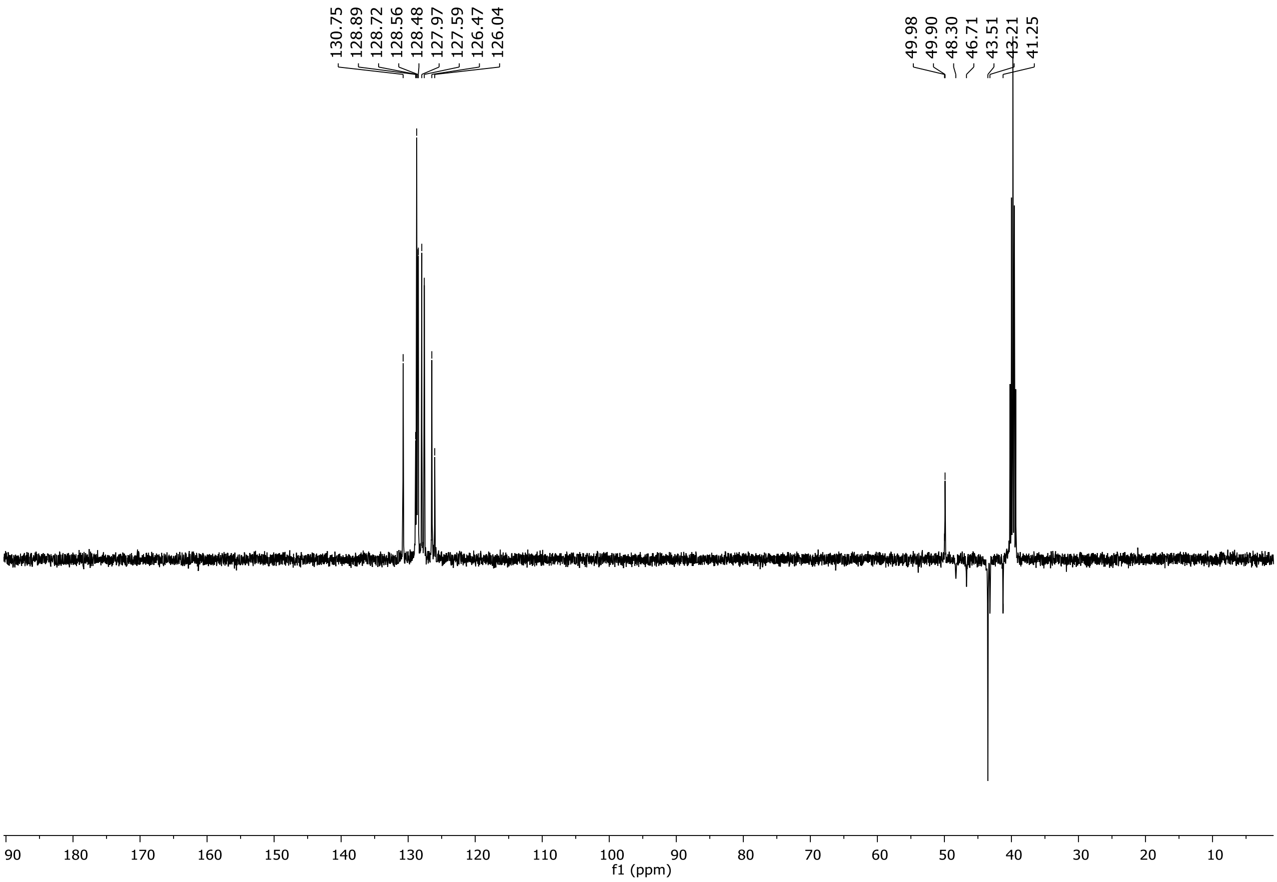

6 (HMBC ^{1}H-^{13}C NMR, 400 MHz, DMSO-d$_6$, 298 K)

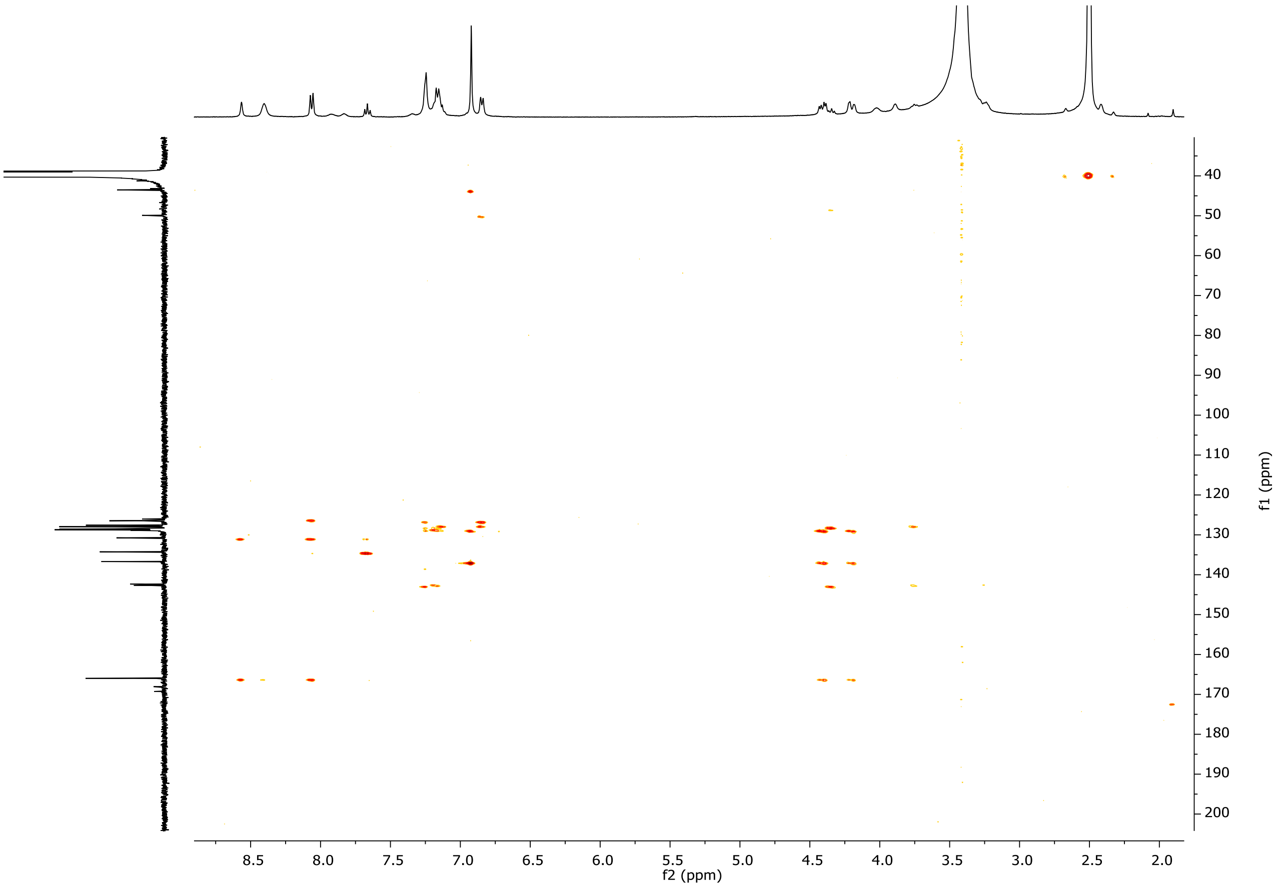

9 (^{1}H NMR, 400 MHz, $CDCl_3$, 298K)

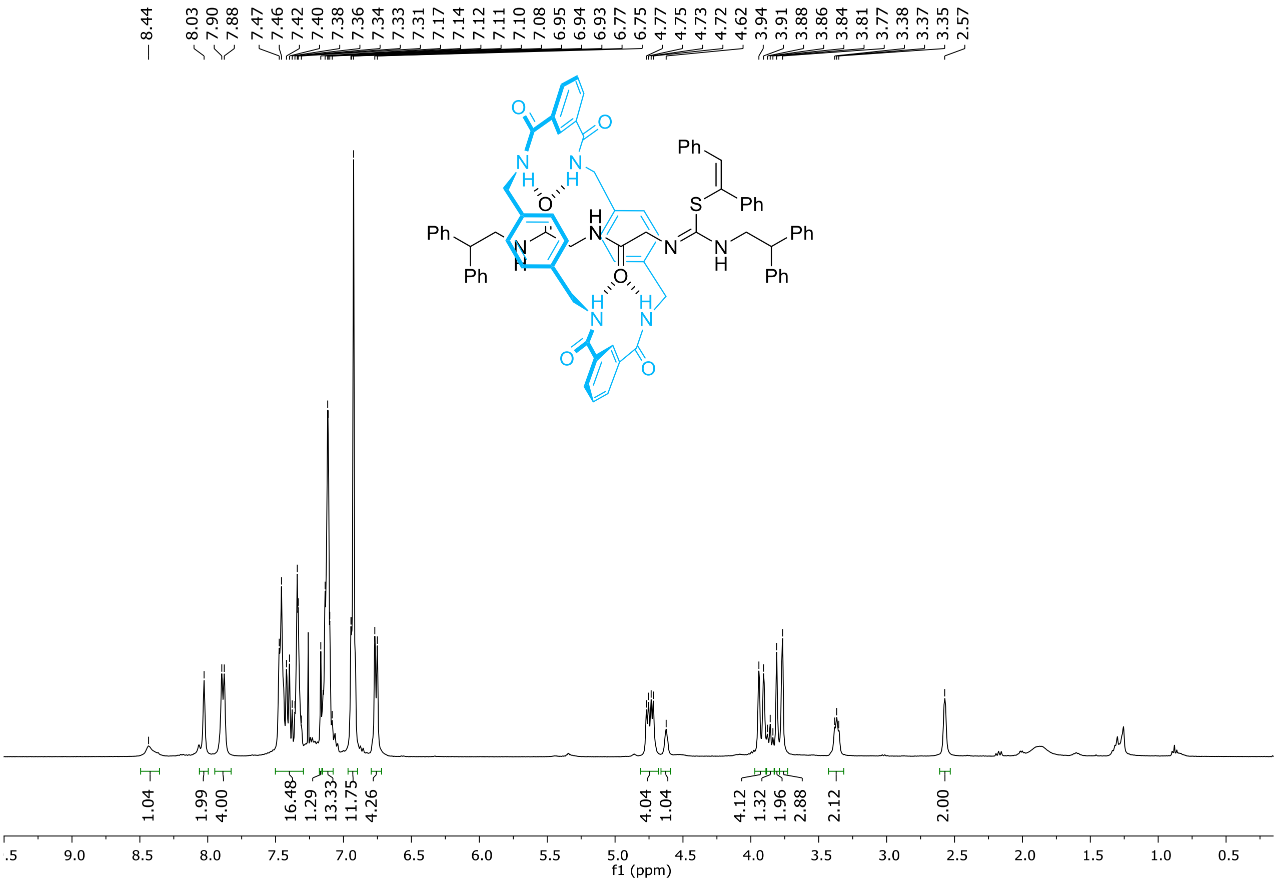

9 (COSY ^{1}H-^{1}H NMR, 400 MHz, $CDCl_3$, 298K)

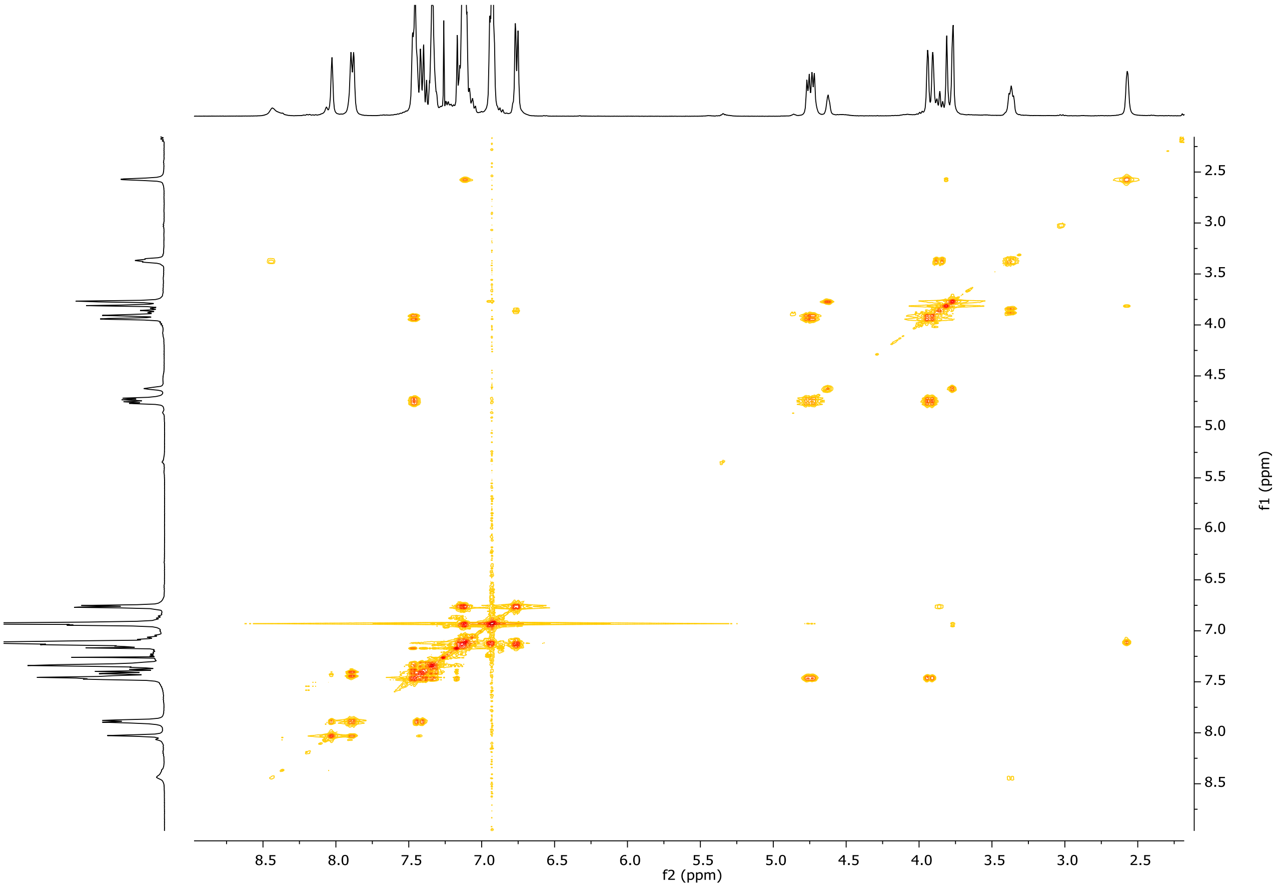

9 (NOESY ^{1}H-^{1}H NMR, 400 MHz, $CDCl_3$, 298K)

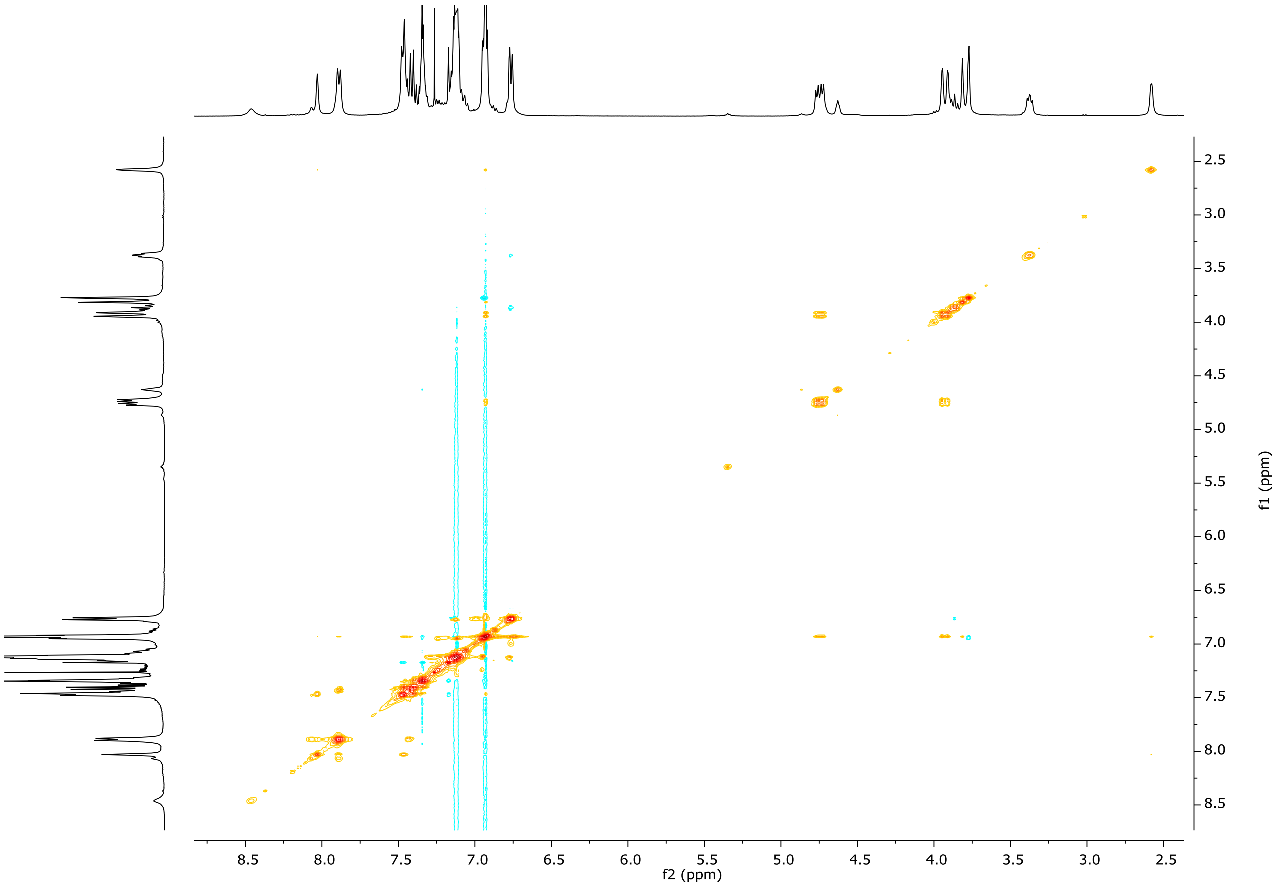

9 (ROESY ^{1}H-^{1}H NMR, 400 MHz, $CDCl_3$, 298K)

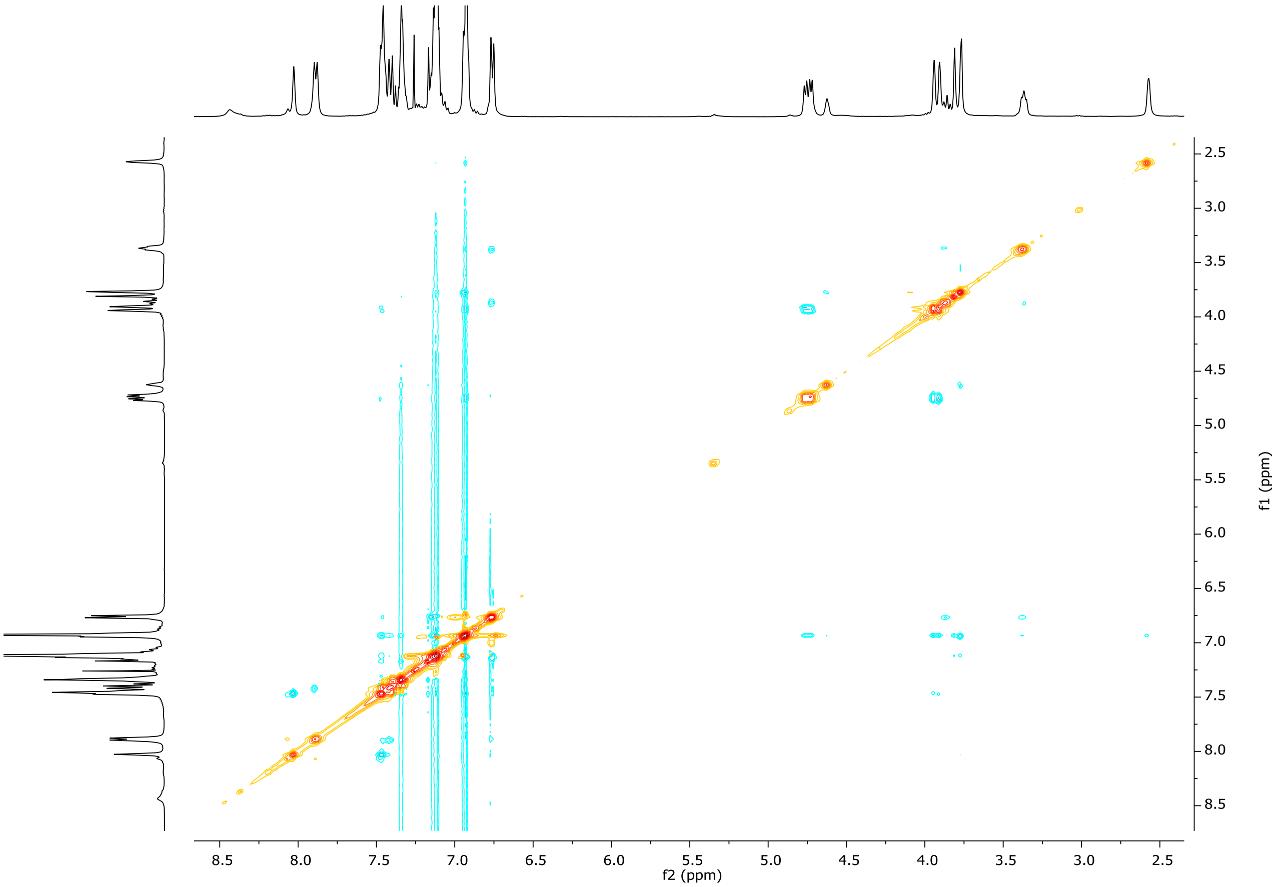

9 (^{13}C NMR, 400 MHz, CDCl$_3$, 298K)

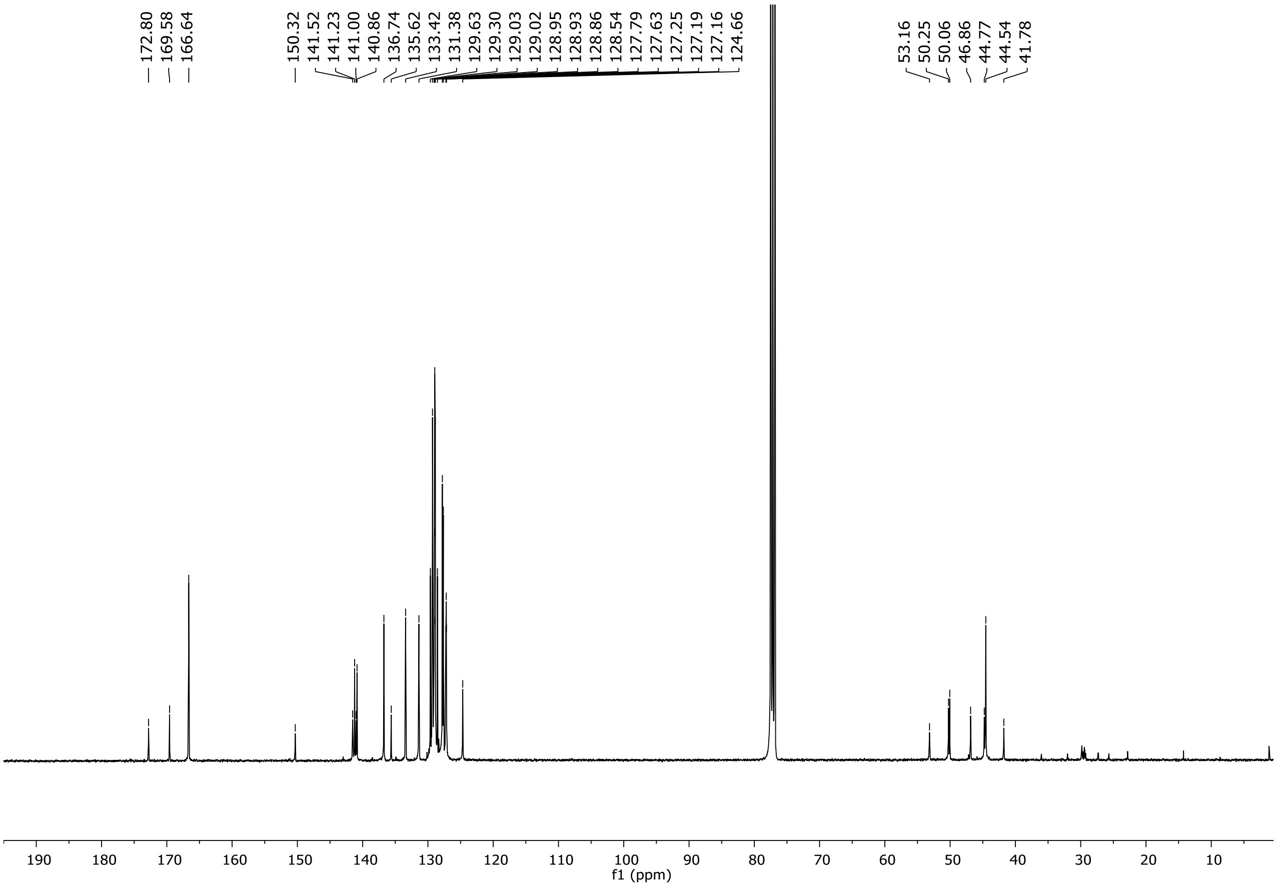

9 (DEPT ^{13}C NMR, 400 MHz, CDCl$_3$, 298K)

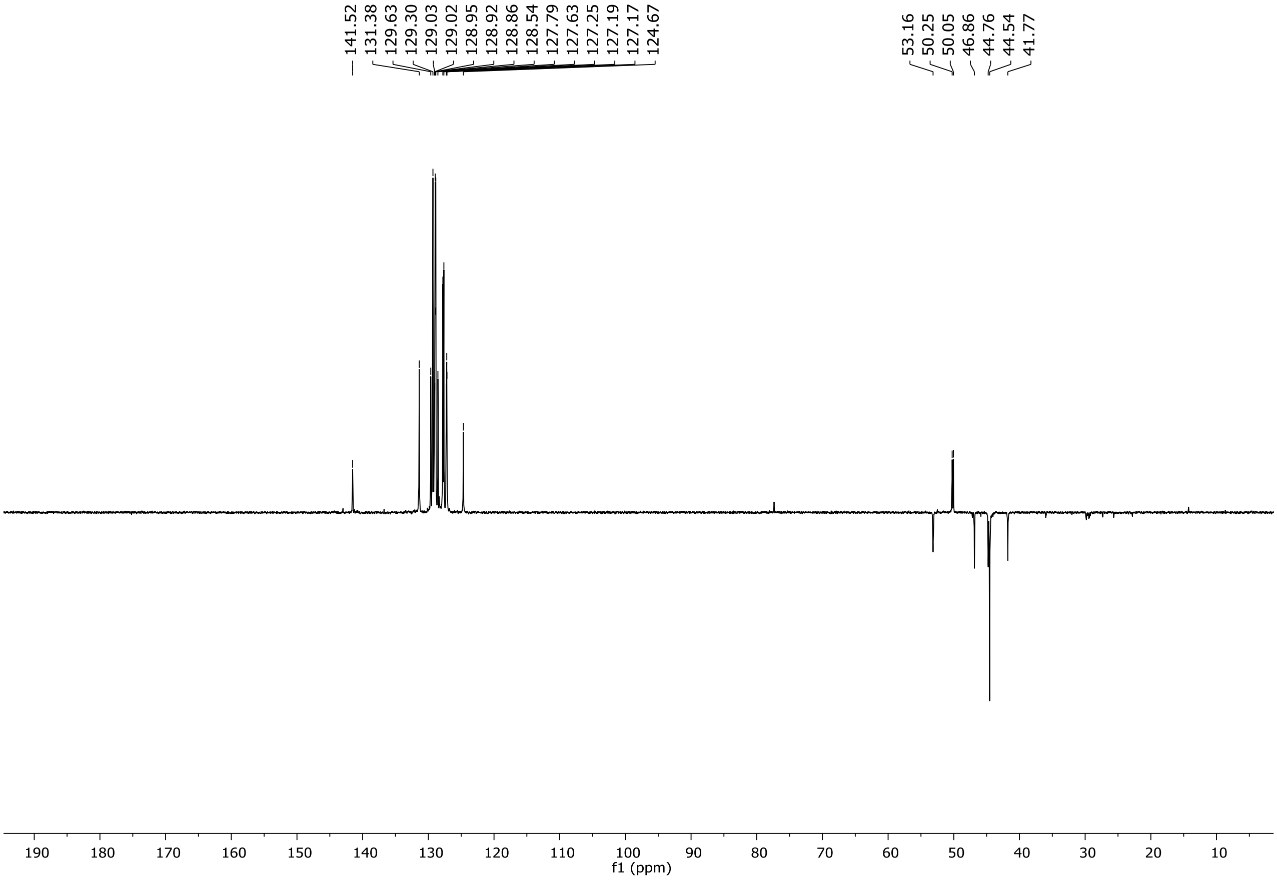

9 (HSQC ^{1}H-^{13}C NMR, 400 MHz, $CDCl_3$, 298K)

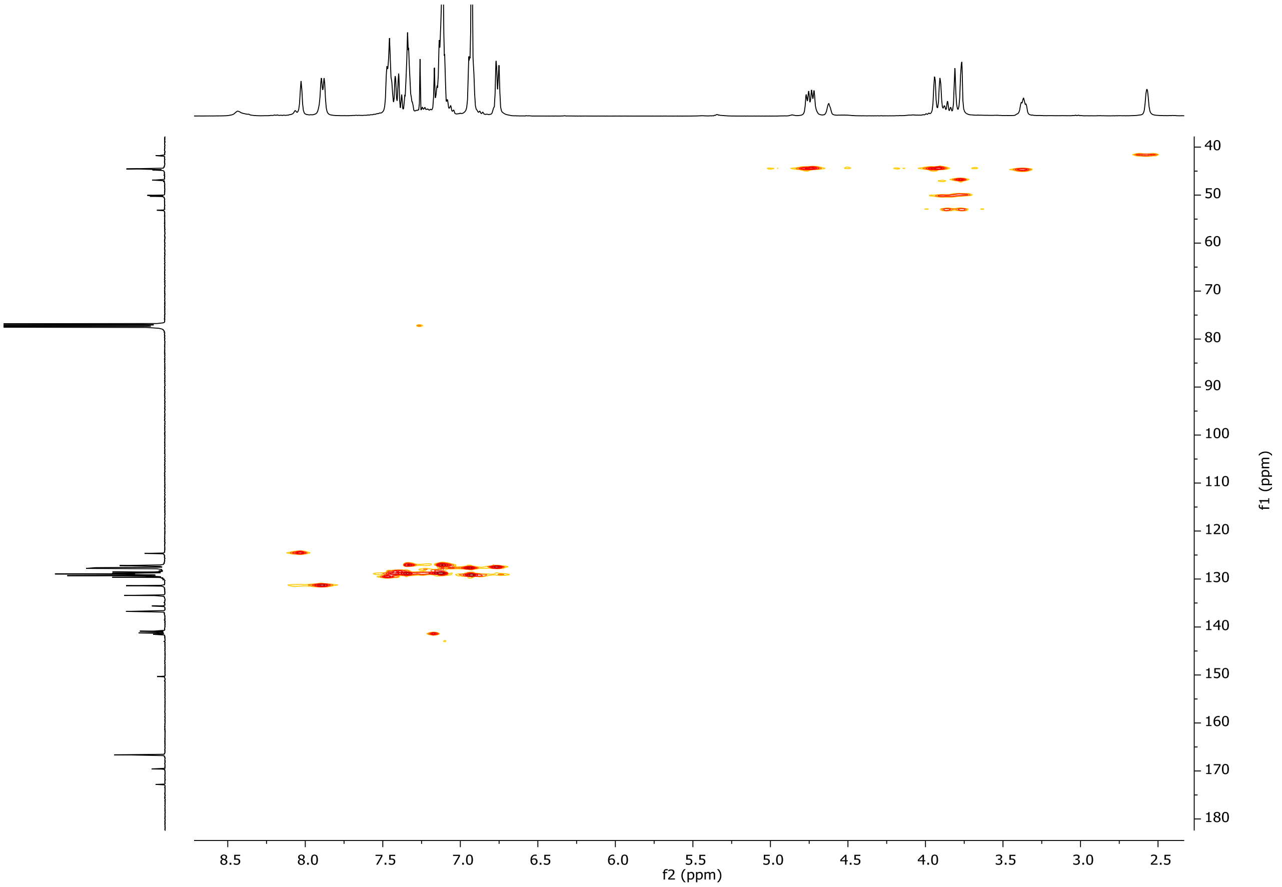

9 (HMBC ^{1}H-^{13}C NMR, 400 MHz, $CDCl_3$, 298K)

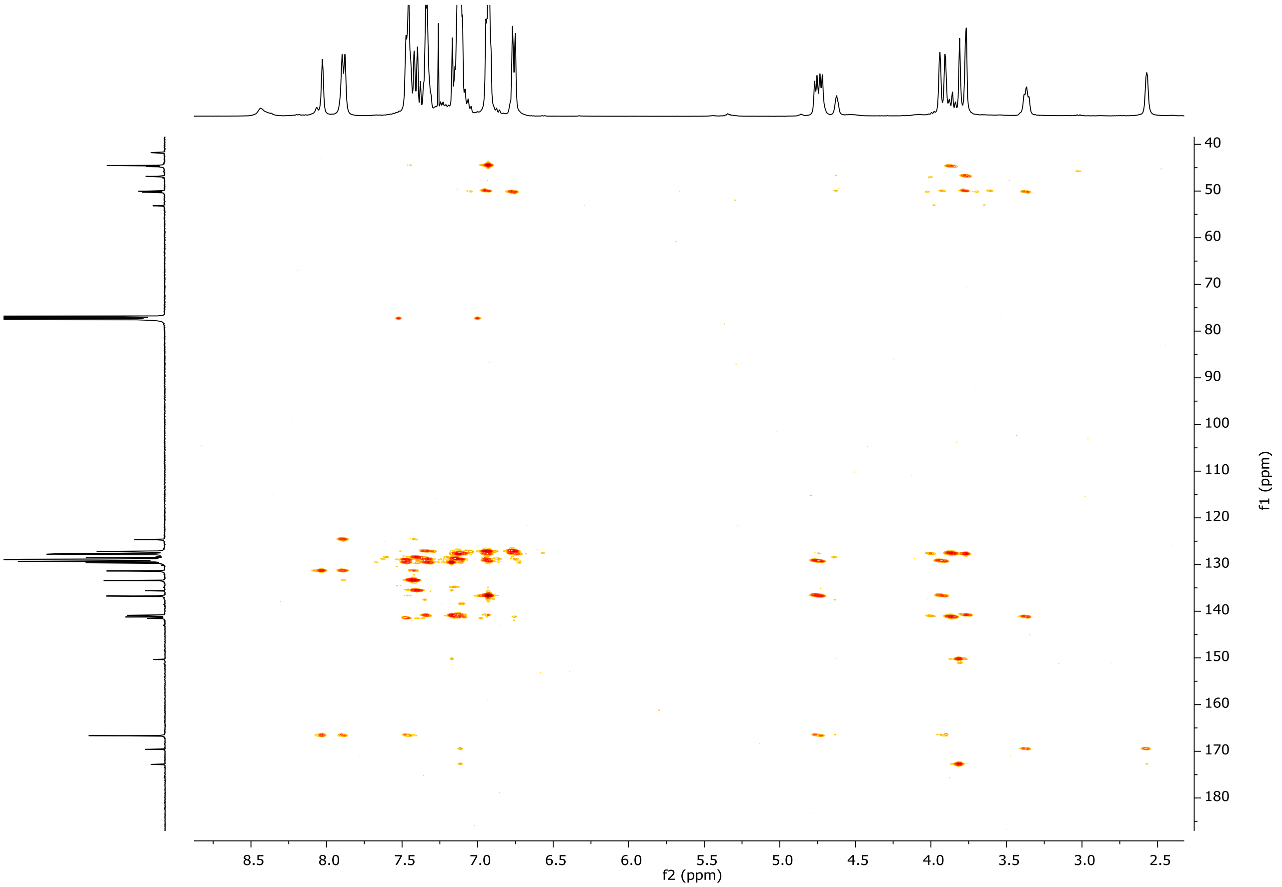

7 (^{1}H NMR, 300 MHz, $CDCl_3$, 298 K)

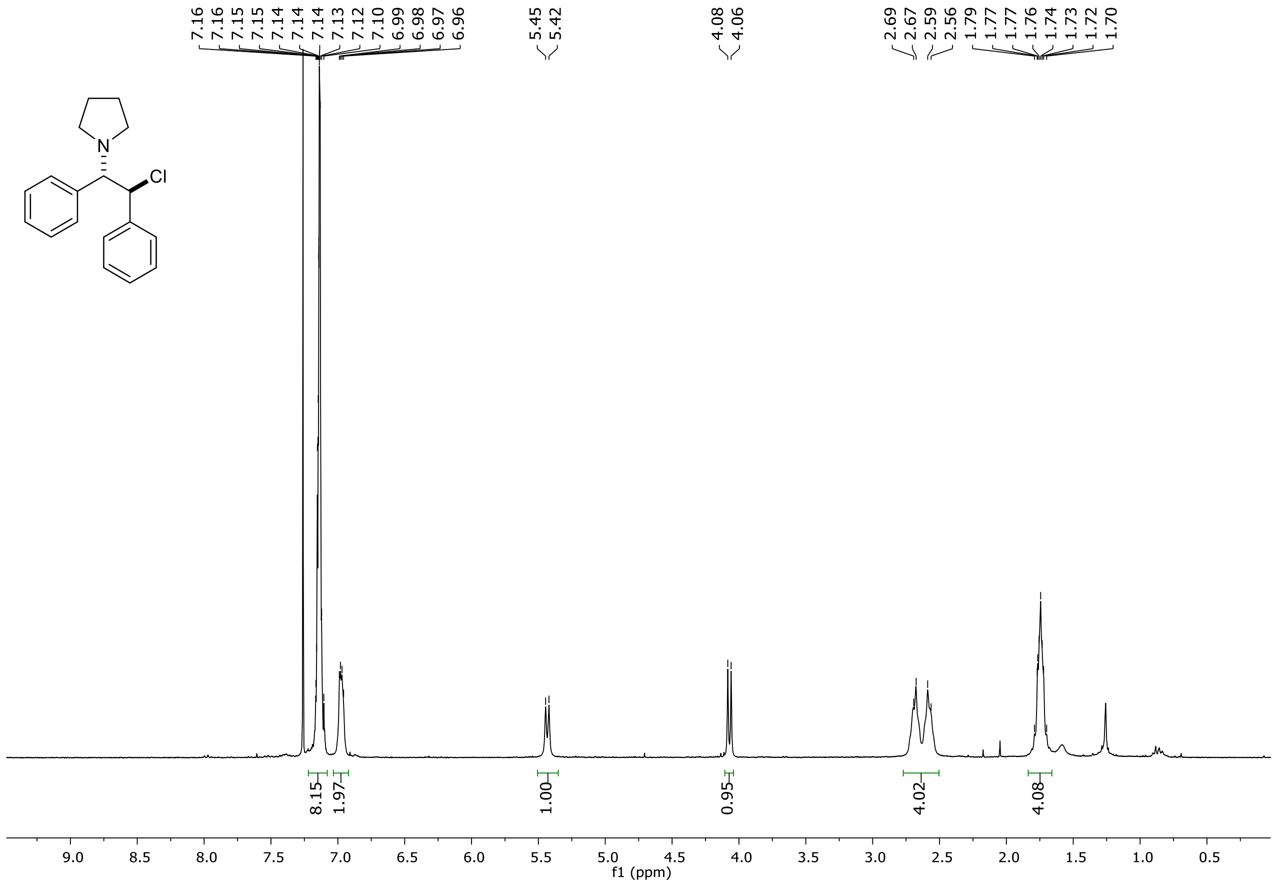

8 (^{1}H NMR, 400 MHz, $CDCl_3$, 298K)

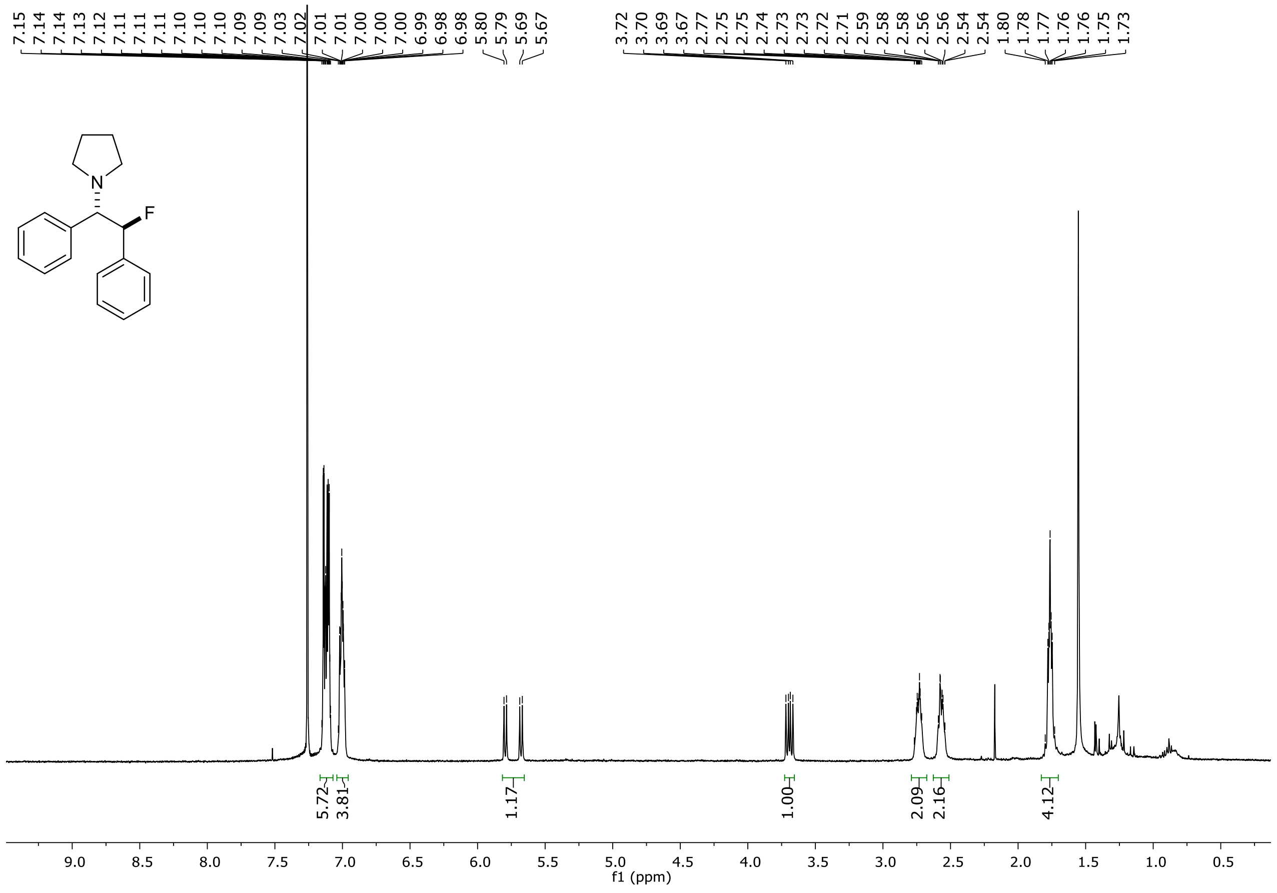

8 (^{19}F NMR, 376 MHz, $CDCl_3$, 298 K)

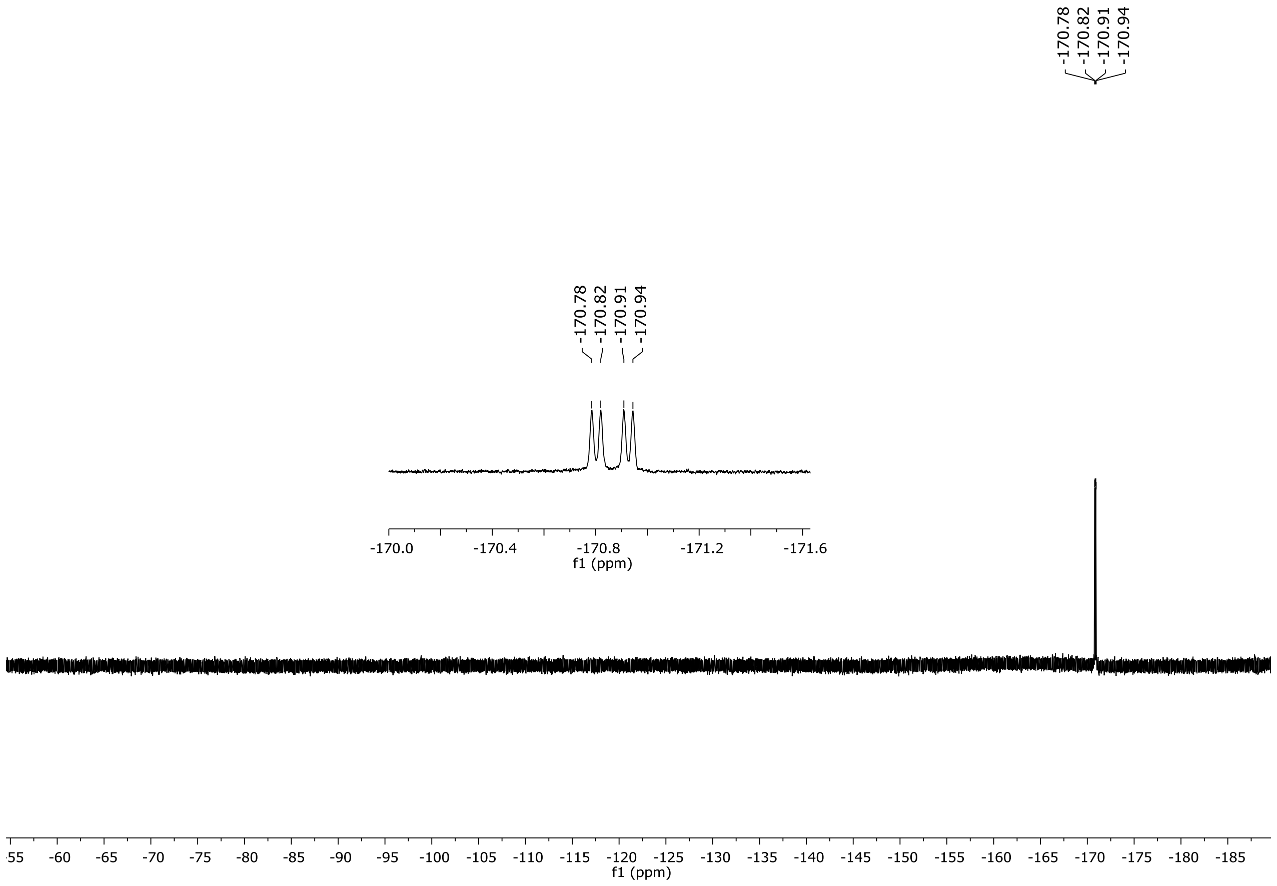

14. References

[1] A. Martinez-Cuezva, F. Morales, G. R. Marley, A. Lopez-Lopez, J. C. Martinez-Costa, D. Bautista, M. Alajarin, J. Berna, *Eur. J. Org. Chem*. **2019**, 3480 – 3488.
[2] M. D. Weingarten, K. Sekanina, W. C. Still, *J. Am. Chem. Soc*. **1998**, *120*, 9112 – 9113.
[3] C. I. Ciobanu, I. Berladean, E. L. Epure, A. Simion, G. Lisa, Y. Boussoualem, I. Carlescu, *Crystals* **2021**, *11*, 1215.
[4] R. P. Megens, T. A. van den Berg, A. Dowine de Bruijn, B. L. Feringa, G. Roelfes, *Chem. Eur. J*. **2009**, *15*, 1723 – 1733.
[5] G. Pupo, A. C. Vicini, D. M. H. Ascough, F. Ibba, K. E. Christensen, A. L. Thompson, J. M. Brown, R. S. Paton, V. Gouverneur, *J. Am. Chem. Soc.* **2019**, *141*, 2878 – 2883.
[6] O. V. Dolomanov, L. J. Bourhis, R. J. Gildea, J. A. K. Howard, H. Puschmann, *J. Appl. Cryst.* **2009**, *42*, 339 – 341.
[7] A. Altomare, G. Cascarano, C. Giacovazzo, A. Guagliardi, *J. Appl. Cryst*. **1993**, *26*, 343.
[8] a) G. M. Sheldrick, F2 SHELXL-2014/7: Program for the Solution of Crystal Structures; University of Göttingen: Göttingen, Germany, **2014**; b) G. M. Sheldrick, *Acta Cryst.* **2015**, *C71*, 3 – 8.
